# Modulation of azole sensitivity and filamentation by *GPI15*, encoding a subunit of the first GPI biosynthetic enzyme, in *Candida albicans*

**DOI:** 10.1038/s41598-019-44919-4

**Published:** 2019-06-11

**Authors:** Priyanka Jain, Pramita Garai, Subhash Chandra Sethi, Nilofer Naqvi, Bhawna Yadav, Pravin Kumar, Sneh Lata Singh, Usha Yadav, Shilpi Bhatnagar, Rahul, Niti Puri, Rohini Muthuswami, Sneha Sudha Komath

**Affiliations:** 1 School of Life Sciences, Jawaharlal Nehru University, New Delhi, 110067 India; 2 Present Address: Post-doctoral Fellow, Fungal Research Group, Institute of Medical Sciences, University of Aberdeen, Aberdeen, UK; 3 Present Address: Research associate, National Institute of Plant Genome Research, New Delhi, 110067 India

**Keywords:** Glycobiology, Fungal biology

## Abstract

Glycosylphosphatidylinositol (GPI)-anchored proteins are important for virulence of many pathogenic organisms including the human fungal pathogen, *Candida albicans*. GPI biosynthesis is initiated by a multi-subunit enzyme, GPI-*N-*acetylglucosaminyltransferase (GPI-GnT). We showed previously that two GPI-GnT subunits, encoded by *CaGPI2* and *CaGPI19*, are mutually repressive. *CaGPI19* also co-regulates *CaERG11*, the target of azoles while *CaGPI2* controls Ras signaling and hyphal morphogenesis. Here, we investigated the role of a third subunit. We show that CaGpi15 is functionally homologous to *Saccharomyces cerevisiae* Gpi15. *CaGPI15* is a master activator of *CaGPI2* and *CaGPI19*. Hence, *CaGPI15* mutants are azole-sensitive and hypofilamentous. Altering *CaGPI19* or *CaGPI2* expression in *CaGPI15* mutant can elicit alterations in azole sensitivity via *CaERG11* expression or hyphal morphogenesis, respectively. Thus, *CaGPI2* and *CaGPI19* function downstream of *CaGPI15*. One mode of regulation is via H3 acetylation of the respective GPI-GnT gene promoters by Rtt109. Azole sensitivity of GPI-GnT mutants is also due to decreased H3 acetylation at the *CaERG11* promoter by Rtt109. Using double heterozygous mutants, we also show that *CaGPI2* and *CaGPI19* can independently activate *CaGPI15*. *CaGPI15* mutant is more susceptible to killing by macrophages and epithelial cells and has reduced ability to damage either of these cell lines relative to the wild type strain, suggesting that it is attenuated in virulence.

## Introduction

GPI anchored proteins play important roles as adhesion molecules, enzymes, activation antigens, differentiation markers, and protozoan coat components. The GPI anchor is synthesized in the endoplasmic reticulum of eukaryotes by the concerted action of multiple enzymes in a pathway that is sequential for the most part^1^. The pathway is essentially conserved in all eukaryotic organisms and generates a glycolipid anchor of the form NH_2_CH_2_CH_2_PO_4_H-6Manα1-2Manα1-6Manα1-4GlcNα1-6D-myo-inositol-1-HPO_4_-lipid, where the lipid can be diacylglycerol, alkylacylglycerol or ceramide. Yet, several species-specific variations in the GPI biosynthetic pathway exist^1,2^.

The process starts with the transfer of *N*-acetylglucosamine (GlcNAc) from UDP-GlcNAc to phosphatidylinositol (PI), a reaction that is in all probability catalyzed by Gpi3 (yeast) or Pig-A (mammals). With the exception of *Giardia lamblia*, in all other eukaryotic systems, Gpi3/Pig-A is assisted by several other proteins that number anywhere between 3 to 6, depending upon the organism, and which together form the GPI-*N*-acetylglucosaminyl transferase (GPI-GnT) complex in the endoplasmic reticulum^3^. In humans, the GPI-GnT complex includes, besides Pig-A, five other subunits, Pig-H, Pig-C, Pig-P, Pig-Q and Pig-Y^1^. Of these, homologs of Pig-C, Pig-P and Pig-Q are present in all eukaryotes. In yeast, they correspond to Gpi2, Gpi19 and Gpi1, respectively. *PIG-Y* is predicted to be homologous to yeast *ERI1*, although the latter could not functionally complement Daudi cells defective in *PIG-Y*^4^. *PIG-H* or yeast *GPI15* is absent in *Caenorhabditis elegans* and *Entamoeba histolytica*^3^. In yeast, deletion of all other than *GPI1* and *ERI1*, are lethal^5,6^.

*C*. *albicans* is an opportunistic pathogen that causes severe invasive as well systemic infections in immunocompromised individuals often leading to mortality. GPI anchored proteins in this organism are important for yeast-to-hyphae transition as well as for virulence^7,8^. Disrupting the GPI biosynthetic pathway results in lethality^9,10^ suggesting that GPI biosynthesis is essential in the organism. In the first set of reports on the GPI-GnT complex of *C*. *albicans*, we showed the importance of *CaGPI19* in growth, drug response and hyphal morphogenesis of this organism^11,12^. The *CaGPI19* deficient mutant was azole sensitive and hyperfilamentous^11^. A mutual co-regulation existed between *CaGPI19* and *CaERG11*, an important gene in the ergosterol biosynthetic pathway and the target of azoles^12^.

We then went on to show that a mutant in a second subunit, *CaGPI2*, was azole resistant and hypofilamentous. Further, *CaGPI2* specifically controlled hyphal morphogenesis *via* Ras signaling. It was also negatively co-regulated with *CaGPI19*^13^.

In the present manuscript, we have explored the role of a third subunit, *CaGPI15*, in *C*. *albicans*. We show that *CaGPI15* is important for growth, cell wall integrity and GPI biosynthesis in *C*. *albicans*. It also affects response to azole drugs as well as hyphal morphogenesis in *C*. *albicans*. It does so because it simultaneously activates both *CaGPI2* and *CaGPI19* which function downstream of *CaGPI15*. Thus, here too CaGpi2 controls hyphal morphogenesis *via* CaRas1 and CaGpi19 controls sensitivity to azoles by regulating *CaERG11* levels. The downregulation of *CaERG11* in mutants of *CaGPI15* as well as *CaGPI19* occurs due to decrease in H3 acetylation on the promoter of *CaERG11*. Both *CaGPI2* and *CaGPI19* can also independently activate *CaGPI15* levels.

## Results

### Cloning of *CaGPI15* gene from *C*. *albicans*

The putative *CaGPI15* gene was identified using human *PIG-H* gene as the query sequence for BLAST analysis as well as using the information available at Prof. Eisenhaber’s website as explained in Materials and Methods. The sequence obtained also compared very well with that reported previously^14^. The putative CaGpi15 protein showed roughly 26.23% and 21.94% identity with Gpi15 sequences from *Saccharomyces cerevisiae* and *Saccharomyces pombe*, respectively (Supplementary Fig. 1A). The gene was subsequently cloned from the genomic DNA of *C*. *albicans* using gene-specific primers.

### *CaGPI15* gene complements the *S. cerevisiae GPI15* gene

The *ScGPI15* gene of YPH500 was placed under the control of the *GAL1* promoter. This strain (YPH-*pGAL1*-*ScGPI15*) grew well in the presence of galactose but was unable to grow in glucose (Supplementary Fig. 1B). When *CaGPI15* was introduced in this strain (YPH-*pGAL1*-*ScGPI15*-*CaGPI15*), the strain was able to grow in glucose (Supplementary Fig. 1B). Growth complementation was accompanied by restoration of GPI-GnT activity (Supplementary Fig. 1C). Thus, despite low sequence conservation, CaGpi15 is the functional homolog of ScGpi15.

### Chromosomal disruption of *CaGPI15* gene

Heterozygous (*CaGPI15Hz*) and conditional null (*Cagpi15 null*) mutants of *CaGPI15* were generated in the *C*. *albicans* BWP17 strain using a PCR based approach^15,16^. *CaGPI15Hz* had one allele of *CaGPI15* disrupted with a *HIS1* nutritional marker^17^. *Cagpi15 null* strain was made in the *CaGPI15Hz* background with the second *CaGPI15* allele placed under the control of the repressible *MET3* promoter. Since *URA3* is known to alter gene expressions in *C*. *albicans*^18^, one copy of *URA3* was inserted at the *RPS1* locus in BWP17 (BWP17URA3) as well as in *CaGPI15Hz* (*CaGPI15Hz-URA3*) and these were used as controls in studies on all mutants that involved use of *URA3* as a selection marker. The downregulation of *CaGPI15* expression levels were confirmed by transcript level analysis (Supplementary Fig. 2A).

### Depletion of *CaGpi15* affects growth of *C*. *albicans*

**Figure 1:**
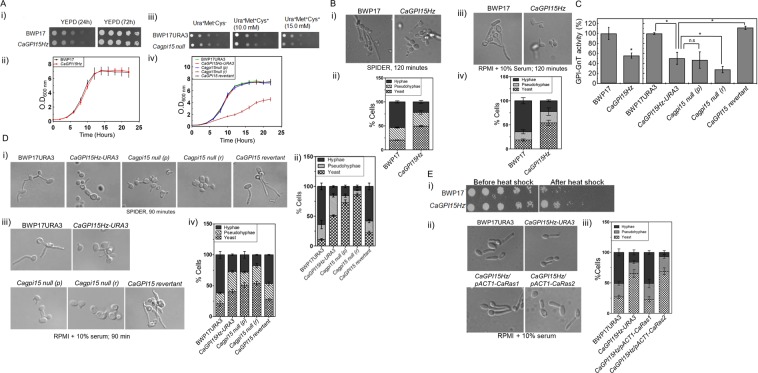
(**A**) *CaGPI15Hz* and *Cagpi15* conditional null mutant show growth defect. (i) BWP17 and *CaGPI15Hz* were spotted on YEPD plates. Growth was monitored at 30 °C for 24 h and 72 h. (ii) *CaGPI15Hz* mutant did not show any growth defect in liquid SD medium. (iii) BWP17URA3 as well as *Cagpi15 null* were spotted on SD medium plates in the absence or presence of Met/Cys. Growth was monitored at 30 °C for 24 h. (iv) *Cagpi15 null* mutant shows growth defect in liquid cultures. *Cagpi15 null* was grown both in absence (p) and presence (r) of 10 mM Met/Cys in liquid medium. For liquid cultures, cell growth for the various strains was monitored by OD_600nm_ at different time points and doubling times are calculated and mentioned in Supplementary Table 2. The experiment was done three times in duplicates; arithmetic mean with standard deviations is shown. For solid media experiments, a 5 µl suspension of cells corresponding to 1 × 10^7^, 2 × 10^6^, 4 × 10^5^, 8 × 10^4^ and 1.6 × 10^4^ numbers were spotted from left to right in each row. The experiments were done thrice using independent cultures. (**B**) *CaGPI15* is required for filamentation. The hyphal growth and quantification of hyphal growth in *CaGPI15Hz* for up to 120 min in (i,ii) liquid spider media and in (iii,iv) liquid RPMI with 10% serum at 37 °C. A minimum of 100 cells were used for the statistical analysis. The arithmetic mean with standard deviation is plotted. (**C**) *CaGPI15* depletion reduces GPI-GnT activity. GPI-GnT activity was tested in the *CaGPI15* mutants as described in Materials and Methods. (**D**) Hyphal growth in *Cagpi15 null* and *CaGPI15* revertant. The hyphal growth and quantification of hyphal growth in *Cagpi15 null* and *CaGPI15* revertant for up to 90 min in (i,ii) liquid spider media and in (iii,iv) liquid RPMI with 10% serum at 37 °C. A minimum of 100 cells were used for the statistical analysis. The arithmetic mean with standard deviations is plotted. (**E**) CaRas1 is responsible for the filamentation phenotypes of the *CaGPI15* mutants. (i) Cells of the indicated strains were spotted on SD-agar plates and incubated at 30 °C for 48 h after a 10 min heat shock at 48 °C; (ii) and (iii) The hyphal growth and quantification of hyphal growth in various strains for up to 120 minutes in liquid RPMI with 10% serum media at 37 °C. CaRas1 restores filamentation in *CaGPI15Hz*. These experiments were done twice in duplicates.

The growth of *CaGPI15Hz*, on solid and liquid medium was comparable to that of the wild type strain (Fig. 1A(i,ii)). The *Cagpi15 null* on the other hand, grew slower on solid minimal media containing Met/Cys (Fig. 1A(iii)). Further, in liquid medium, the doubling time for the *Cagpi15 null* in the presence of 10 mM Met/Cys was found to be higher than in the absence of Met/Cys (Fig. 1A(iv); Supplementary Table 2).

### Depletion of *CaGpi15* results in reduced GPI-GnT activity in *C*. *albicans*

We previously showed that the GPI-GnT activity was reduced in the *CaGPI15Hz* strain^17^. Not only was the GPI-GnT activity significantly lower in the *CaGPI15Hz* (~50%) with or without the *URA3* marker as compared to the wild type controls it was further reduced in the *Cagpi15 null* strain (28% activity) under repressive conditions of growth (Fig. 1C). Under permissive growth conditions in the *Cagpi15 null* there is no significant decrease in the GPI-GnT activity (47%) as compared to *CaGPI15Hz-URA3* (Fig. 1C). That a drop in GPI-GnT activity of roughly 50% does not seem to cause a corresponding reduction in growth of the strain would suggest that relatively low levels of GPI biosynthetic activity are sufficient for the growth of *C*. *albicans*. This has also been reported in other GPI biosynthetic mutants^9,11,13,19^. However, when GPI biosynthesis drops below a certain threshold, as is seen in the conditional null strain under repressive growth conditions, then it affects the growth of the fungus. GPI-GnT activity was restored in *CaGPI15* revertant strain where one allele of *CaGPI15* was reintroduced in the *CaGPI15Hz* using the constitutively active *pACT1* promoter (Fig. 1C).

### Depletion of *CaGpi15* causes cell wall defects

The *CaGPI15* knock-down mutants showed several cell wall defects, including increased clumping when grown to near saturation levels and lower chitin and beta glucan levels in the cell wall versus the wild type strain (Supplementary Fig. 2B,C; Supplementary Table 3). The cell wall defects were reversed in the *CaGPI15* revertant strain (Supplementary Fig. 2B,C(i–iv)), suggesting that the cell wall defects were specifically due to depletion of CaGpi15.

### *CaRas1*-dependent signaling pathway is altered in the *CaGPI15* mutants

As also shown previously^17^, hyphal growth of *CaGPI15Hz* was noticeably lesser than that of the wild type BWP17 strain on solid as well as liquid hyphae-inducing media at 37 °C but was restored in the *CaGPI15* revertant cells (Fig. 1B(i–iv); Fig. 1D(i–iv); Supplementary Fig. 3A), suggesting that this effect was specific to *CaGPI15*.

We have previously shown that the Ras/cAMP dependent PKA activity is altered in mutants of the first step of GPI anchor biosynthesis in *C albicans*^12,13,17^. Hyperactive Ras mutants are heat shock sensitive^13,20^ and reduced Ras signaling correlates with heat-shock resistance^13^. *CaGPI15Hz* was resistant to heat shock as compared to BWP17 (Fig. 1E(i)), suggesting that Ras-dependent cAMP/PKA signaling was decreased in this mutant. *C*. *albicans* has two Ras proteins, CaRas1 and CaRas2 of which CaRas1 is known to be the major determinant of hyphal growth^21^. Overexpression of *CaRAS1* restores filamentation in *CaGPI5Hz* while overexpression of *CaRAS2* does not (Fig. 1E(ii,iii); Supplementary Fig. 3B).

### *CaGPI15* mutant strains are sensitive to azoles due to compromised ergosterol biosynthesis

**Figure 2:**
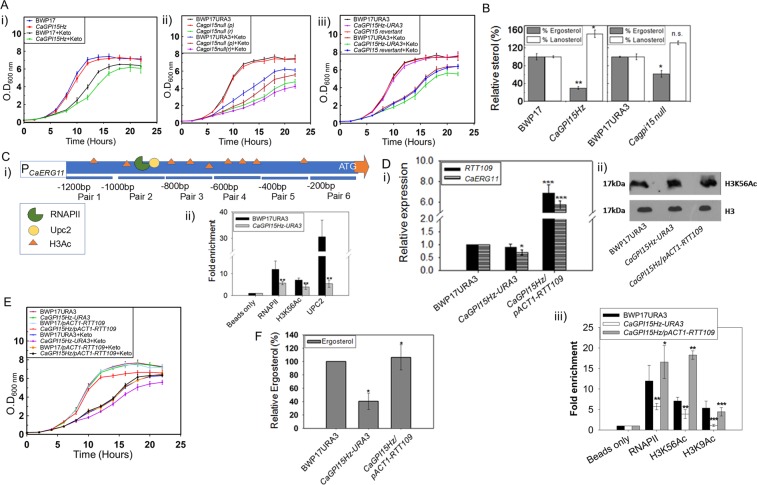
Ergosterol biosynthesis pathway is affected in the *CaGPI15* mutants. (**A**) *CaGPI15* mutants are sensitive to ketoconazole and the sensitivity is reversed in the *CaGPI15 revertant* in liquid SD medium. (i) Growth curve analysis of *CaGPI15Hz* mutant with respect to BWP17 in 0.08 μg/ml ketoconazole. Growth of the strains without ketoconazole are also shown for comparison. (ii) *Cagpi15 null* mutant was grown both in permissive as well as in repressive liquid medium in the presence of 0.2 μg/ml ketoconazole. (iii) Azole sensitivity is reversed in the *CaGPI15* revertant strain in the presence of 0.2 μg/ml ketoconazole. Supplementary Table 2 contains the doubling time for the mutants both in the presence/absence of ketoconazole. The experiments are done twice in duplicates and the arithmetic mean and standard deviations are plotted. (**B**) *CaGPI15* mutants accumulate lanosterol and are deficient in ergosterol. Relative quantification of ergosterol (grey bars) and lanosterol (white bars) was done by GC-MS. (**C**) Rtt109 regulates *CaERG11* transcription in the *CaGPI15Hz*. (i) Schematic representation of the *CaERG11* promoter pairs. Pair 2 shows the binding site for RNA Pol II and Upc2. H3K56Ac sites are also identified on the promoter. (ii) ChIP analysis was done to compare the occupancy of RNA pol II, H3K56Ac and Upc2 at *CaERG11* promoter using primer pair 2 in *CaGPI15Hz*-*URA3* relative to BWP17URA3. (**D**) Levels of *CaERG11* are restored in *CaGPI15Hz*/*pACT1*-*RTT109* strain. (i) Transcript levels of *CaERG11* is restored in *CaGPI15Hz*/*pACT1*-*RTT109* strain. The transcript levels of both *RTT109* and *CaERG11* were monitored in the mutants relative to BWP17URA3. (ii) The H3K56Ac levels in whole cell lysate were estimated in the mutants relative to BWP17URA3. H3 levels were taken as a loading control. A cropped representative image is shown, and the full-length image is displayed in Supplementary Fig. 5D (iii) The *CaERG11* promoter occupancy of RNA pol II, H3K56Ac and H3K9Ac in the mutants relative to BWP17URA3. (**E**) Azole sensitivity was reversed in *CaGPI15Hz*/*pACT1*-*RTT109* strain. Growth curve analysis of *CaGPI15Hz/pACT1-RTT109* mutant in 0.2 μg/ml ketoconazole. The doubling time of *CaGPI15Hz*/*pACT1*-*RTT109* in the presence of ketoconazole is almost similar with the wildtype strain grown in the same condition (Supplementary Table 2). (**F**) *CaGPI15Hz*/*pACT1*-*RTT109* strain accumulates ergosterol. Relative quantification of ergosterol was done using GC-MS. P-values for *CaGPI15Hz* were calculated relative to the wild type while for *CaGPI15Hz*/*pACT1*-*RTT109* it was relative to *CaGPI15Hz*-*URA3*.

*CaGPI15Hz* as well as *Cagpi15 null* cells were sensitive to azoles as compared to controls (Fig. 2A(i,ii); Supplementary Fig. 4A(i,ii)). This sensitivity was reversed in the *CaGPI15* revertant strain (Fig. 2A(iii); Supplementary Fig. 4A(iii)). Azoles target CaErg11, the lanosterol demethylase in the ergosterol biosynthetic pathway of *C*. *albicans*^22^. Hence, the levels of *CaERG11* transcripts in cells of *CaGPI15Hz* and *Cagpi15 null* were examined. *CaERG11* levels were significantly reduced in both cases (Supplementary Fig. 4B(i)). *CaERG11* levels were restored in the *CaGPI15* revertant strain (Supplementary Fig. 4B(ii)). The reduction in *CaERG11* levels correlated well with the accumulation of lanosterol, the substrate of CaErg11, and a reduction in total ergosterol levels in the *CaGPI15Hz* and *Cagpi15 null* strains (Fig. 2B).

Upc2 is a transcription factor that controls *CaERG11* levels in the cell^23^. The occupancy of both RNA Pol II (RNAPII) as well as Upc2 were significantly reduced on the promoter of *CaERG11* in *CaGPI15Hz-URA3* as compared to BWP17URA3 when probed using primer pair 2 (Fig. 2C(i,ii)), suggesting that the promoter had reduced accessibility for transcription. This also correlated well with the fact that acetylation of histone H3, as assessed using the H3K56 antibody, was significantly lower on the promoter of *CaERG11* in *CaGPI15Hz-URA3* as compared to that in BWP17URA3 (Fig. 2C(ii) & Supplementary Fig. 4D).

The role of histone acetylation in the regulation of *CaERG11* expression in *C*. *albicans* was further investigated. H3K56 and H3K9 acetylation are mediated by Rtt109, a histone acetyltransferase^24,25^. As can be seen from (Fig. 2D(i,ii); Supplementary Fig. 5D), the expression of *RTT109* at the mRNA as well as protein levels was not significantly altered in the *CaGPI15Hz* strain. *RTT109* was then expressed under the control of *pACT1* promoter in the *CaGPI15Hz* strain and its overexpression was confirmed (Fig. 2D(i)). *CaERG11* transcript levels were upregulated in these cells (Fig. 2D(i)). ChIP analysis showed that overexpression of *RTT109* in *CaGPI15Hz* cells increased acetylation of H3, as assessed by anti-H3K56 or anti-H3K9 antibodies, and increased the occupancy of RNAPII on the *CaERG11* promoter (Fig. 2D(iii)) Overexpression of *RTT109* in *CaGPI15Hz* cells also reversed the azole sensitivity (Fig. 2E; Supplementary Fig. 4C) and restored ergosterol levels in these cells (Fig. 2F), confirming that acetylation of H3 by Rtt109 regulates the expression of *CaERG11* in this strain.

Separately, in order to ensure that the reduced H3 acetylation was not due to a global reduction in acetylation or due to defects in nucleosome assembly, we checked the H3 acetylation levels at two different intergenic regions (Chromosome 5 and Chromosome R) in BWP17URA3, *CaGPI15Hz-URA3* and *CaGPI15Hz-pACT1-RTT109* by ChIP (Supplementary Fig. 4E(i,ii). No reduction in H3 acetylation was observed in the two strains at these positions relative to the control, BWP17URA3, suggesting that the ChIP signal observed is not a nucleosome assembly dependent effect and was instead due to a reduction in acetylation levels at the promoter of *CaERG11*.

The acetylation mediated by Rtt109 is catalyzed with two histone chaperones, Vps75 and Asf1^26^. While the latter has been shown to be important for H3K56Ac alone, the former has been shown to be important for acetylation of H3 at K9 as well K56^25^. A *VPS75Hz* mutant should, therefore, mimic a strain defective in acetylation by Rtt109. To confirm that acetylation of H3 at the *CaERG11* promoter was important for its regulation in *C*. *albicans*, ChIP analysis was done in a *VPS75Hz* strain generated in the lab. The acetylation of H3 dropped to a significant extent at the promoters of *CaERG11*, *CaGPI2*, *CaGPI15* and *CaGPI19* in this strain when probed using anti-H3K56 antibody (Supplementary Fig. 4F(i–iv)).

### *CaGPI2* and *CaGPI19* levels in the *CaGPI15* heterozygous strain can also be restored by *RTT109* overexpression

**Figure 3:**
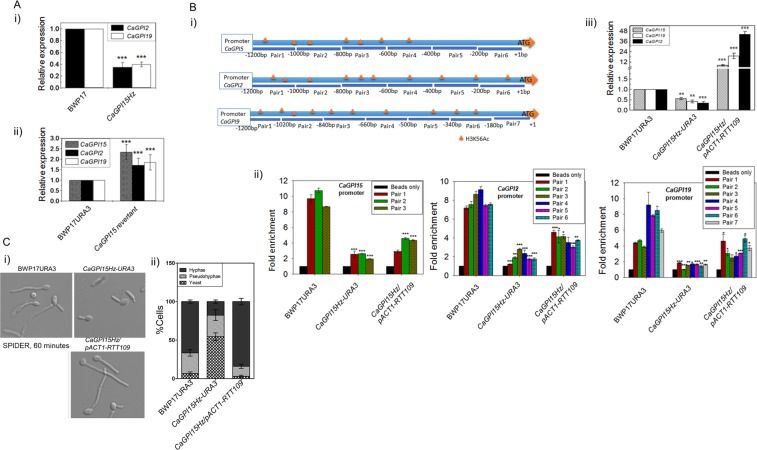
*CaGPI2* and *CaGPI19* are downstream of *CaGPI15*. (**A**) Transcript levels of *CaGPI2* and *CaGPI19* are controlled by *CaGPI15*. (i) The transcript levels of both *CaGPI2* and *CaGPI19* in *CaGPI15Hz* as compared to BWP17. (ii) The transcript levels of *CaGPI15*, *CaGPI2* and *CaGPI19* in the *CaGPI15* revertant. P-values were calculated with respect to the wild type controls. (**B**) H3K56Ac and transcript levels of *CaGPI2*, *CaGPI15* and *CaGPI19* are controlled by Rtt109. (i) Schematic representation of the promoters of *CaGPI15*, *CaGPI2* and *CaGPI19* along with their H3K56Ac positions. (ii) The relative H3K56Ac levels at the promoters of *CaGPI15*, *CaGPI2* and *CaGPI19* in the indicated strains versus wild type. (iii) Transcript levels of *CaGPI15*, *CaGPI19* and *CaGPI2* upon *RTT109* overexpression in the *CaGPI15Hz*. **(C**) Hyphal morphogenesis is restored upon overexpression of *RTT109*. (i) and (ii) The hyphal growth and quantification of hyphal growth in various strains for up to 60 minutes in liquid spider media at 37 °C. A minimum of 100 cells were used for the statistical analysis. The experiment was repeated twice in duplicates; averages with standard deviations are shown.

Transcript levels of both *CaGPI2* and *CaGPI19* were found to be reduced in *CaGPI15Hz* as compared to BWP17 (Fig. 3A(i)) and were restored in the *CaGPI15* revertant strain (Fig. 3A(ii)), suggesting that this effect was specifically linked to *CaGPI15* levels. It should be noted that the expression of two housekeeping genes (*CaUBC13*, *CaACT1*), two ergosterol biosynthesis genes downstream to *CaERG11* (*CaERG3*, *CaERG4*) and three other downstream GPI biosynthetic genes (*CaGPI12*, *CaGPI14*, *CaGPI8*) were all found to not be significantly altered in the *CaGPI15Hz* or the *CaGPI15* revertant strains (Supplementary Fig. 5A).

Further, the acetylation of histone H3 on the promoter of *CaGPI15*, *CaGPI2* and *CaGPI19* in the *CaGPI15Hz* strain was found to be significantly reduced when probed using anti-H3K56 antibody (Fig. 3B(i,ii)). Overexpression of *RTT109* in *CaGPI15Hz* restored the H3Ac (Fig. 3B(ii)) and the levels of all three genes (Fig. 3B(iii). It also reversed azole sensitivity as described above (Fig. 2E; Supplementary Fig. 4C), and filamentation of the *CaGPI15Hz* mutant (Fig. 3C(i,ii)). Taken together, these results indicate that *CaGPI15* specifically regulates *CaGPI19* and *CaGPI2* levels.

### *CaGPI2* and *CaGPI19* control the phenotypes of *CaGPI15*

**Figure 4:**
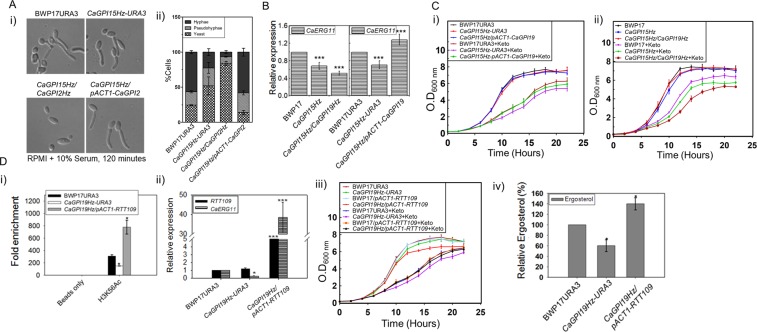
**(A**) Hyphal morphogenesis correlates with *CaGPI2* expression levels. (i) and (ii) The hyphal growth and quantification of hyphal growth in various strains for up to 120 minutes in liquid RPMI with 10% serum media at 37 °C. A minimum of 100 cells were used for the statistical analysis. The experiment was repeated twice in duplicates; averages with standard deviations are shown. (**B**) *CaERG11* transcript levels correlate with levels of *CaGPI19* expression. The transcript levels of *CaERG11* were monitored in the mutants strains relative to either BWP17 or BWP17URA3. (**C**) The azole response of the strains correlates with *CaERG11* expression levels. (i) Strains overexpressing *CaGPI19* were grown in liquid SD medium containing 0.2 µg/ml ketoconazole. (ii) Depletion of *CaGPI19* slows down the doubling time of the *CaGPI15Hz/CaGPI19Hz* mutant when cultured with 0.08 µg/ml ketoconazole. Supplementary Table 2 contains the doubling time for the mutants both in the presence/absence of ketoconazole. Two independent experiments in duplicates were done for confirmation. (**D**) Rtt109 regulates *CaERG11* transcription and sensitivity to azoles in the *CaGPI19* mutant. (i) ChIP analysis was done to compare the H3K56Ac of *CaERG11* promoter in the mutant strains relative to BWP17URA3. (ii) Transcript levels of *RTT109* and *CaERG11* were monitored in the mutant strains relative to BWP17URA3. (iii) Response to azoles was monitored in *CaGPI19Hz/pACT1-RTT109* mutant. All strains were grown with or without 0.2 μg/ml ketoconazole and the doubling time was calculated (Supplementary Table 2). The experiments were repeated twice in duplicates. (iv) Relative quantification of ergosterol was done in different mutants of *CaGPI19* using GC-MS. P-values for all heterozygous mutants were calculated with respect to the wild type controls while that for overexpression mutants are with respect to *CaGPI19Hz-URA3*.

We recently demonstrated that *CaGPI2* functions downstream of *CaGPI15* in controlling hyphal morphogenesis^17^. Filamentation of two strains, *CaGPI15Hz/CaGPI2Hz* and *CaGPI15Hz/pACT1-CaGPI2*, confirmed this. In the former, where one allele of *CaGPI2* is disrupted in *CaGPI15Hz* background, filamentation was further reduced (Fig. 4A(i,ii); Supplementary Fig. 3C). In the latter, where *CaGPI2* was overexpressed in the *CaGPI15Hz* background, filamentation was restored (Fig. 4A(i,ii); Supplementary Fig. 3C). Similarly, to confirm that *CaGPI19* functions below *CaGPI15* in controlling *CaERG11* levels and sensitivity to azole drugs, *CaGPI15Hz/CaGPI19Hz* and *CaGPI15Hz/pACT1-CaGPI19* strains were generated. In the former, one allele of *CaGPI19* was disrupted and in the latter *CaGPI19* was overexpressed in the *CaGPI15Hz* background. *CaERG11* transcripts were reduced in *CaGPI15Hz/CaGPI19Hz* and upregulated in *CaGPI15Hz/pACT1-CaGPI19* as compared to the parent strain (Fig. 4B). The sensitivities of these strains to azoles also correlated with their *CaERG11* levels. *CaGPI15Hz/CaGPI19Hz* was more sensitive to azoles while *CaGPI15Hz/pACT1-CaGPI19* was resistant to azoles as compared to *CaGPI15Hz* (Fig. 4C(i,ii); Supplementary Fig. 5B). Thus, *CaGPI19* functions downstream of *CaGPI15* in controlling sensitivity to azole drugs.

ChIP analysis showed that acetylation of H3 was reduced at the promoter of *CaERG11* in *CaGPI19Hz* strain and was restored by overexpression of *RTT109* in *CaGPI19Hz* background (Fig. 4D(i)). Overexpression of *RTT109* in *CaGPI19Hz* background also caused an enhancement in *CaERG11* transcripts (Fig. 4D(ii)), reversal of azole sensitivity (Fig. 4D(iii); Supplementary Fig. 5C and restoration in ergosterol levels (Fig. 4D(iv)).

### The cross-talk between *CaGPI2*, *CaGPI15* and *CaGPI19*

**Figure 5:**
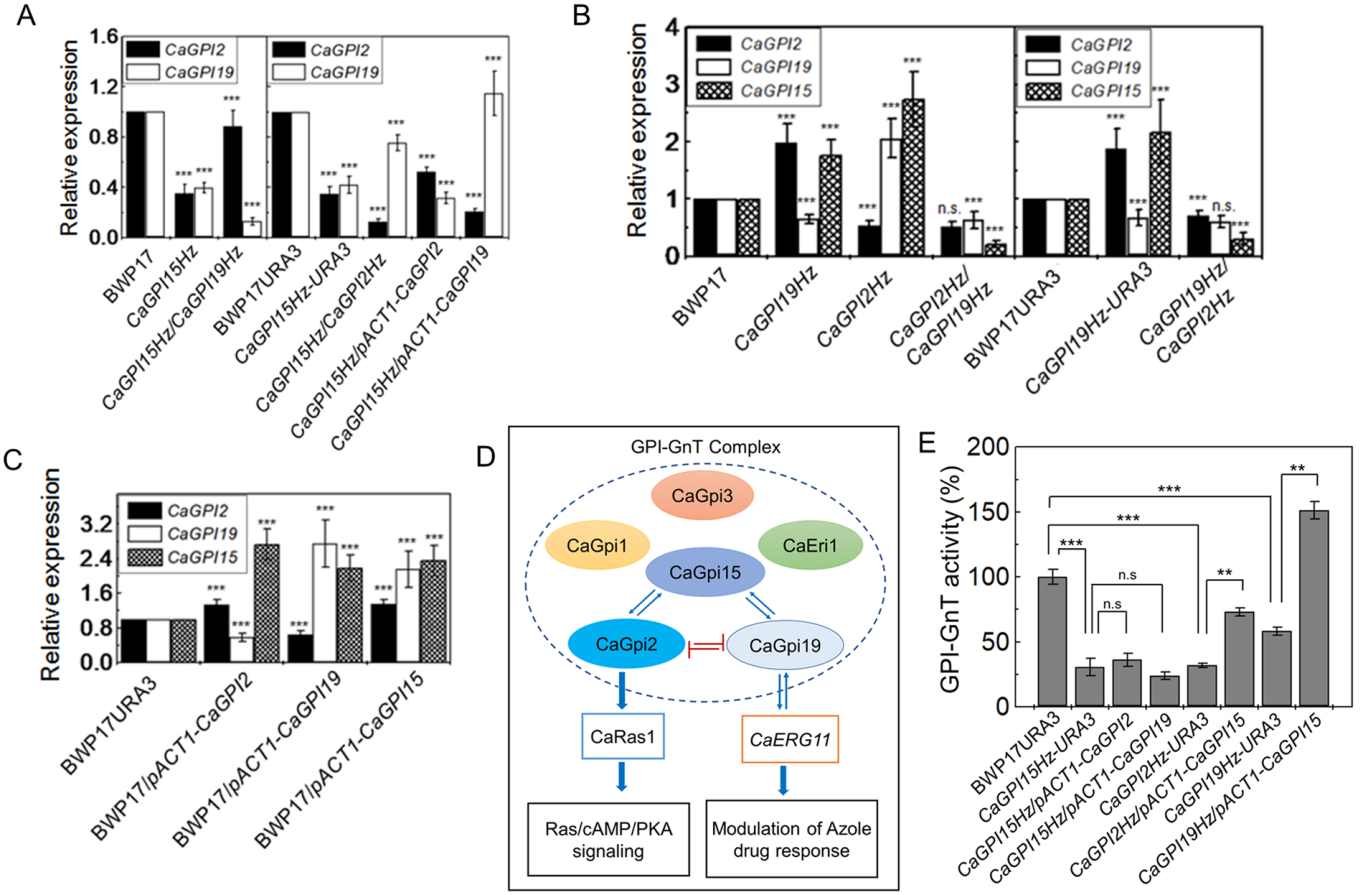
Once activated *CaGPI19* and *CaGPI2* are not sensitive to depletion of *CaGPI15*. (**A**) The mutual negative regulation between *CaGPI2* and *CaGPI19* is maintained in *CaGPI15* mutants. Heterozygous deletion of either *CaGPI2* or *CaGPI19* was made in *CaGPI15Hz*. Likewise, either *CaGPI2* or *CaGPI19* was overexpressed in *CaGPI15Hz*. The transcript levels of *CaGPI19* and *CaGPI2* were monitored in these mutants. (**B**) Simultaneous downregulation of *CaGPI2* and *CaGPI19* downregulates *CaGPI15*. Transcript levels of *CaGPI2*, *CaGPI19* and *CaGPI15* were monitored in double heterozygote strains of *CaGPI2* and *CaGPI19* along with their respective heterozygous parent strains. (**C**) Activation of *CaGPI15* correlates with activation of either *CaGPI2* or *CaGPI19*. Transcript levels of *CaGPI2*, *CaGPI19* and *CaGPI15* were monitored in BWP17 strain overexpressing either *CaGPI2*, *CaGPI19* or *CaGPI15* relative to BWP17URA3. (**D**) Model explaining the regulation of *CaERG11* transcription and filamentation by *CaGpi15*. In *C*. *albicans*, *CaGPI15* is an activator of *CaGPI2* as well as *CaGPI19*. In turn, both *CaGPI2* and *CaGPI19* are independently capable of stimulating activation of *CaGPI15*. *CaGPI2* and *CaGPI19* are mutually negatively regulated and function downstream of *CaGPI15* to control Ras signaling and *CaERG11* levels, respectively, in the organism. Azole response *via* regulation of *CaERG11* continues to correlate with *CaGPI19* levels while Ras1-dependent hyphal morphogenesis continues to correlate with *CaGPI2* levels. (**E**) *CaGPI15* can mediate active GPI-GnT complex formation in *CaGPI2Hz* and *CaGPI19Hz* strains. Relative GPI-GnT activity in *CaGPI15Hz* strain overexpressing either *CaGPI2* or *CaGPI19* as well as in *CaGPI2Hz* and *CaGPI19Hz* strains overexpressing *CaGPI15*. P-values for heterozygous mutants were calculated with respect to the wild type controls while that for double deletion or overexpression mutants was calculated relative to the heterozygous parent control.

The data presented in Fig. 3A, suggested that *CaGPI15* is an activator of both *CaGPI2* and *CaGPI19*. To examine the interaction between *CaGPI2* and *CaGPI19* in the *CaGPI15Hz* background, we studied two double heterozygous strains, *CaGPI15Hz/CaGPI2Hz* and *CaGPI15Hz/CaGPI19Hz*. The levels of *CaGPI19* were increased in *CaGPI15Hz/CaGPI2Hz* while that of *CaGPI2* were increased in *CaGPI15Hz/CaGPI19Hz*. Overexpressing either *CaGPI2* or *CaGPI19* in *CaGPI15Hz* resulted in downregulation of the other (Fig. 5A). Thus, the mutually negative regulation between *CaGPI2* and *CaGPI19* continues to function in the *CaGPI15Hz* strain.

*CaGPI15* transcripts are upregulated in *CaGPI2Hz*^13^ and *CaGPI19Hz* (Fig. 5B). We also examined two strains that had simultaneous downregulation of *CaGPI2* and *CaGPI19*. Transcripts of *CaGPI15* decreased in both *CaGPI19Hz/CaGPI2Hz* and *CaGPI2Hz/CaGPI19Hz* strains (Fig. 5B). Overexpression of *CaGPI15* in the wild type strain led to simultaneous upregulation of *CaGPI2* and *CaGPI19* (Fig. 5C). Similarly, overexpression of either *CaGPI2* or *CaGPI19* caused overexpression of *CaGPI15* while maintaining the negative regulation between *CaGPI2* and *CaGPI19* (Fig. 5C). Thus, both *CaGPI2* and *CaGPI19* can independently activate *CaGPI15* (Fig. 5D). *CaGPI15* acts upstream and serves to activate both *CaGPI2* and *CaGPI19* (Fig. 5D).

GPI-GnT activity assays also corroborate such a model. Overexpression of *CaGPI15* in *CaGPI2Hz* or *CaGPI19Hz* pushes up transcript levels of both *CaGPI2* and *CaGPI19* and causes enhanced GPI-GnT activity in comparison to the parent strains (Fig. 5E). However, due to the negative regulation between *CaGPI2* and *CaGPI19*, overexpressing either *CaGPI2* or *CaGPI19* in the *CaGPI15Hz* strain cannot enhance its GPI-GnT activity (Fig. 5E).

### *CaGPI15* heterozygous strain is more susceptible to killing by MH-S macrophage cells and is less able to kill the macrophage cell line

**Figure 6:**
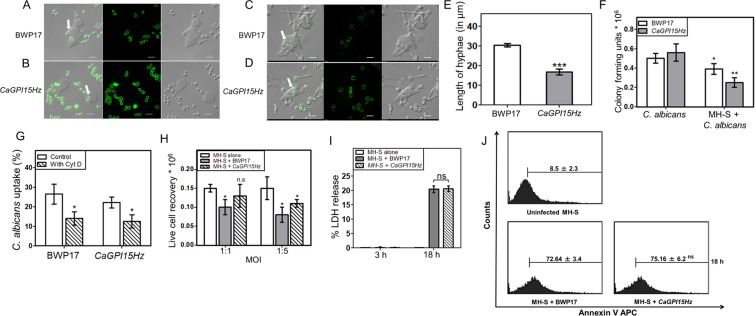
Comparative virulence study of BWP17 and *CaGPI15Hz* with macrophages. Phagocytosis of yeast (**A**,**B**) and Hyphal (**C**,**D**) form of BWP17 and *CaGPI15Hz*, respectively, by MH-S cells. MH-S cells were cultured on coverslips overnight for adherence and then cells were co-cultured with CFSE labelled BWP17 and *CaGPI15Hz* for 3 h (**A**,**B**) & 18 h (**C**,**D**) respectively followed by fixation with PFA and were mounted and examined using a confocal laser scanning microscope (Magnification 100X). Scale bar represents 10 µm. (**E**) Hyphae formation by *C*. *albicans*. The hyphae length was quantified using Nikon NIS element software. (**F**) CFU assay after incubation with MH-S cells. MH-S cells (0.1 million) were seeded in 48 well cell culture plate and kept for adherence followed by addition of the indicated *C*. *albicans* strains at MOI 1:5 for 18 h. The MH-S cells were lysed and plated on YEPD plates kept at 30 °C for 24 h. The colony forming units obtained were quantified and plotted. (**G**) Uptake of BWP17 and *CaGPI15Hz* by MH-S cells. MH-S cells (0.3 million) were seeded in 24 well cell culture plate and allowed to adhere overnight in CO2 incubator at 37 °C. Cells were pre-treated with 2.5 µg/ml cytochalasin D for 30 min followed by addition of CFSE labelled BWP17 and *CaGPI15Hz* at MOI 1:5 for 3 h and processed as discussed in Methods. The uptake of the *C*. *albicans* strains was calculated and plotted on the basis of percentage of cells taking up yeast form of BWP17 and *CaGPI15Hz*. (**H**) Live cell recovery of MH-S. MH-S cells (0.1 million) were seeded in 48 well cell culture plate. After adherence, *C*. *albicans* at MOI 1:1 and MOI 1:5 for 18 h were added to these cultures. Cells were trypsinized, washed and live cell recovery was calculated by trypan blue exclusion method. (**I**) *C*. *albicans* induced MH-S macrophage pyroptosis. MH-S cells (50,000) were seeded in 96 well cell culture plate. BWP17 and *CaGPI15Hz* at MOI 1:5 were added for 3 h and 18 h. 50 µl of supernatant were transferred to another 96 well cell culture plate followed by addition of substrate reagent for 30 min and then the reaction was stopped. (**J**) *C*. *albicans* induced MH-S macrophage apoptosis. MH-S (0.3 million) were seeded in 24 well cell culture plate and allowed to adhere overnight in CO_2_ incubator at 37 °C. BWP17 and *CaGPI15Hz* at MOI 1:5 were added for 3 h and 18 h and processed as discussed in Methods. Cells were harvested and 1 µl Annexin V APC was added. Each point represents mean ± SEM of values obtained from three independent assays. *p ≤ 0.05, **p ≤ 0.005 and ***p ≤ 0.0005 represent statistically significant difference between control and treated cells, ns is no statistically significant difference. The significance of any difference was calculated by using one-tailed distribution in a two-sample equal variance student’s t test.

We tested the effect of *CaGPI15* knock-down on *C*. *albicans* virulence. For this, a murine alveolar macrophage cell line (MH-S) was co-cultured with BWP17 and *CaGPI15Hz* strain for 3 h (Fig. 6A,B) and 18 h (Fig. 6C,D). Both strains of *Candida* formed hyphae when co-cultured with MH-S cells for 18 h. BWP17 co-cultured with MH-S had 55% longer hyphae than *CaGPI15Hz* co-cultured with MH-S for 18 h (Fig. 6E). The internalization of *C*. *albicans* cells by MH-S was roughly similar for the *CaGPI15Hz* and BWP17 strains (Fig. 6F). This was also the case when phagocytosis was inhibited with the help of cytochalasin D (Cyt D) (Fig. 6F). Thus, mutation of *CaGPI15* does not appear to alter the phagocytosis of *C*. *albicans* cells by either cytoskeleton-dependent or independent pathways. The colony forming units (CFU) recovered after incubation with the macrophage cells was significantly lower in *CaGPI15Hz* as compared to BWP17 (Fig. 6G). Hence, more numbers of *CaGPI15Hz* cells were killed by MH-S in comparison to BWP17. In addition, more live MH-S cells were recovered after co-culture with *CaGPI15Hz* as compared to BWP17, suggesting that *CaGPI15Hz* was less virulent (Fig. 6H). There was also no difference in cell death by pyroptosis after co-culturing MH-S with BWP17 and *CaGPI15Hz* at 1:5 multiplicity of infection (MOI) for 3 h and 18 h (Fig. 6I). But there is a significant difference in pyroptosis between 3 h and 18 h for MH-S co-cultured with either strain. Similarly, there was no difference in cell death by apoptosis (Annexin V staining) when MH-S cells were co-cultured with either with BWP17 or *CaGPI15Hz* strain at 1:5 MOI for 18 h. Nevertheless, the level of apoptosis was significant in MH-S on infection with either strain of *C*. *albicans* for 18 h. So, *C*. *albicans* seems to cause MH-S macrophage cell death by apoptosis as well as pyroptosis.

### *CaGPI15* heterozygous strain is more susceptible to killing by LA-4 epithelial cells and is less able to kill the epithelial cell line

**Figure 7:**
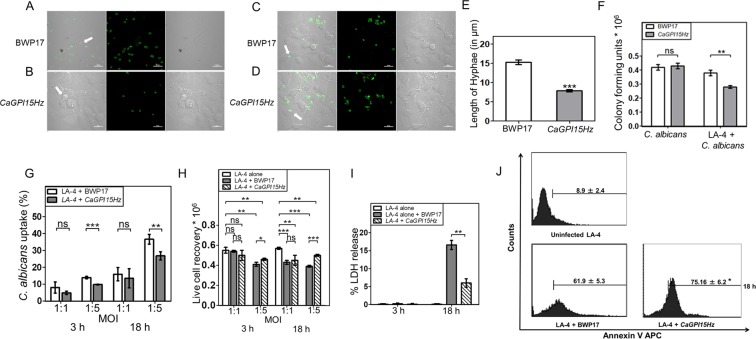
Comparative virulence study of BWP17 and *CaGPI15Hz* with epithelial cells. Phagocytosis of yeast (**A**,**B**) and Hyphal (**C**,**D**) form of BWP17 and *CaGPI15Hz*, respectively, by LA-4 cells. LA-4 cells were cultured on coverslips overnight for adherence and then cells were co-cultured with CFSE labelled BWP17 and *CaGPI15Hz* for 3 h (**A**,**B**) & 18 h (**C**,**D**) respectively followed by fixation with PFA and were mounted and examined using a confocal laser scanning microscope (Magnification 60X). Scale bar represents 20 µm. (**E**) Hyphae formation by *C*. *albicans*. The hyphae length was quantified using Nikon NIS element software. (**F**) CFU assay after incubation with LA-4 cells. LA-4 cells (0.1 million) were seeded in 48 well cell culture plate and kept for adherence followed by addition of the indicated *C*. *albicans* strains at MOI 1:5 for 18 h. The LA-4 cells were lysed and plated on YEPD plates kept at 30 °C for 24 h. The colony forming units obtained were quantified and plotted. (**G**) Uptake of BWP17 and *CaGPI15Hz* by LA-4 cells. LA-4 cells (0.3 million) were seeded in 24 well cell culture plate and allowed to adhere overnight in CO2 incubator at 37 °C. Cells were co-cultured with CFSE labelled BWP17 and *CaGPI15Hz* at MOI 1:1 (as control) & 1:5 for 3 h & 18 h and processed as discussed in Methods. The uptake of the *C*. *albicans* strains was calculated and plotted on the basis of percentage of cells taking up yeast form of BWP17 and *CaGPI15Hz*. (H) Live cell recovery of LA-4 cells. LA-4 cells (0.3 million) were seeded in 24 well cell culture plate. After adherence, *C*. *albicans* at MOI 1:1 & 1:5 for 3 h & 18 h were added to these cultures. Cells were trypsinized, washed and live cell recovery was calculated by trypan blue exclusion method. (**I**) *C*. *albicans* induced LA-4 cells pyroptosis. LA-4 cells (50,000) were seeded in 96 well cell culture plate. BWP17 and *CaGPI15Hz* at MOI 1:5 were added for 3 h and 18 h. 50 µl of supernatant were transferred to another 96 well cell culture plate followed by addition of substrate reagent for 30 min and then the reaction was stopped. (**J**) *C*. *albicans* induced LA-4 cells apoptosis. LA-4 cells (0.3 million) were seeded in 24 well cell culture plate and allowed to adhere overnight in CO_2_ incubator at 37 °C. BWP17 and *CaGPI15Hz* at MOI 1:5 were added for 3 h and 18 h and processed as discussed in Methods. Cells were harvested and 1 µl Annexin V APC was added. Each point represents mean ± SEM of values obtained from three independent assays. *p ≤ 0.05, **p ≤ 0.005 and ***p ≤ 0.0005 represent statistically significant difference between control and treated cells, ns is no statistically significant difference. The significance of any difference was calculated by using one-tailed distribution in a two-sample equal variance student’s t test.

We further investigated the interaction of *CaGPI15Hz* cells with an epithelial cell line. Murine epithelial cell line LA-4 cells were co-cultured with BWP17 and *CaGPI15Hz* strain for 3 h (Fig. 7A,B) and 18 h (Fig. 7C,D). Both strains formed hyphae on co-culture with LA-4 cells for 18 h (Fig. 7C,D) but BWP17 exhibited 51% longer hyphae than *CaGPI15Hz* (Fig. 7E). *CaGPI15Hz* cells were more susceptible to killing by LA-4 cells as significantly lower CFU were obtained for *CaGPI15Hz* co-cultured with LA-4 in comparison to that for BWP17 (Fig. 7F). Phagocytosis of BWP17 cells by LA-4 cells was significantly higher than that of *CaGPI15Hz* cells at MOI 1:5 (Fig. 7G), suggesting a role for fungal GPI anchored proteins in their uptake^27^. However, the live cell recovery of LA-4 cells was significantly higher for those co-cultured with *CaGPI15Hz* versus those with BWP17 at MOI 1:5 (Fig. 7H). No significant difference in phagocytosis or live cell recovery was observed in experiments using either of these strains at MOI 1:1. Significantly lower pyroptosis (~30% of wildtype) was seen in LA-4 cells when co-cultured for 18 h with *CaGPI15Hz* in comparison to those co-cultured with BWP17. No pyroptosis was seen when LA-4 cells were infected with either strain for 3 h. Further, we found a small but significantly higher degree of apoptosis in LA-4 cells infected with BWP17 cells versus *CaGPI15Hz* after incubation for 18 h at 1:5 MOI (Fig. 7J). Hence, the killing induced by *C*. *albicans* infection in epithelial cells also involved apoptosis and pyroptosis. The data from epithelial cell infection studies also supports the notion that the *CaGPI15Hz* strain is attenuated in virulence.

## Discussion

The GPI anchor glycolipid is produced in the endoplasmic reticulum in 10–12 sequential biochemical steps. In lower eukaryotes this pathway is essential to the growth and viability of the organism while in higher eukaryotes it is critical only at certain stages of organismal development, such as in embryogenesis. Given their essentiality for eukaryotic pathogens, several steps of the pathway have been the focus of study as probable drug targets^28,29^. However, isolating and studying the individual enzymes of the GPI biosynthetic pathway is challenging because it involves mostly multi-subunit membrane-bound enzymes of relatively low abundance. No high-resolution X-ray crystallographic data are available for any of the enzymes till date and there are no commercially available substrates for most steps of the pathway. The study of the GPI biosynthetic pathway in *C*. *albicans* is made more challenging due to its codon biasness. *C*. *albicans* proteins heterologously expressed in another host may at times need to be codon optimised for function. Further, until recently no cell free assay system was available for the GPI biosynthetic pathway in this organism since protocols used to generate microsomes from the closely related yeast, *S*. *cerevisiae*, did not yield active microsomes from *C*. *albicans*^13,19^.

Despite the many challenges, studying the GPI anchor biosynthesis of different organisms without depending solely on model organisms can be very rewarding. For example, the first and committed step of the GPI biosynthesis pathway in eukaryotes is an important site for regulation. The presence of poorly conserved accessory subunits to assist the highly conserved catalytic subunit suggests that the regulation of the GPI biosynthetic pathway could be mediated *via* these proteins. Some evidence in support of such a hypothesis also exists. In *S*. *cerevisiae*, Ras2 was shown to inhibit the GPI-GnT complex and *vice versa* while no such regulation was observed in mammals^4,6,30^. Similarly, Dpm2 is known to regulate the mammalian GPI-GnT, but an equivalent regulation is not observed in other organisms^31^. In a series of papers establishing such a link in *C*. *albicans*, we showed that the GPI-GnT complex interacts with and regulates two other important pathways, ergosterol biosynthesis and hyphal morphogenesis in *C*. *albicans*^11–13^. Specifically, CaGpi19 controls *CaERG11* levels and modulates azole drug response while CaGpi2 regulates hyphal morphogenesis by controlling Ras signaling. Moreover, CaRas1, the *C*. *albicans* homolog of *S*. *cerevisiae* Ras2, activates rather than inhibits the GPI-GnT activity^17^. In turn, the CaRas1-dependent PKA signaling pathway is activated by CaGpi2, but this is independent of the GPI-GnT activity itself^17^.

In the present study, we examined a third subunit of the GPI-GnT complex to elucidate its importance for *C*. *albicans*. CaGpi15, like CaGpi2 and CaGpi19, is a poorly conserved protein sharing very low sequence homology with its yeast counterpart. Yet, at the functional level it complements a conditionally lethal *S*. *cerevisiae gpi15* mutant. Thus, significant functional similarities exist between the Gpi15 homologs in the two organisms. In *C*. *albicans*, as in *S*. *cerevisiae*, *CaGPI15* appears to be important for cell growth. Gene-dosage effects also operate here since the heterozygous mutants were only marginally affected in doubling times while the conditional null showed significantly longer doubling times as compared to the wild type strains. Cell wall defects and clumping observed in *CaGPI15* mutants appear to be a result of GPI anchor deficiency since these have also been observed in other GPI biosynthetic mutants^9,11–13^.

*CaGPI15* mutants were ergosterol deficient and sensitive to azoles. Thus, in its response to azoles, it appeared that *CaGPI15* mutants mirrored the *CaGPI19* mutants. Yet, surprisingly, the hyphal morphogenesis phenotype of *CaGPI15* mutant cells was quite unlike the hyperfilamentous phenotype of *CaGPI19* mutants^11,12^. *CaGPI15* mutants were hypofilamentous as compared to the wild type strains due to reduced Ras signaling, a feature also observed in *CaGPI2* mutants^13^. Thus, *CaGPI15* exhibited phenotypes of a mutant that had both *CaGPI2* and *CaGPI19* downregulated. A transcript level analysis confirmed this. Significantly reduced histone H3 acetylation was observed on the promoters of *CaGPI2*, *CaGPI15* and *CaGPI19* in the *CaGPI15* mutant strain. Overexpression of *RTT109* could restore H3Ac as well as the transcript levels of the three genes, suggesting that expression levels of these genes are regulated *via* histone H3 acetylation in the *CaGPI15* mutant strain. Overexpressing *CaGPI15* could also restore *CaGPI2* and *CaGPI19* levels. Phenotypic assays as well as GPI-GnT activity assays corroborated these results.

Using double mutants in which one allele of either *CaGPI2* or *CaGPI19* was disrupted in the heterozygous *CaGPI15* background, we discovered that *CaGPI2* and *CaGPI19* continued to be mutually negatively regulated. Further, the hyphal morphogenesis and azole drug response phenotypes correlated with changes in *CaGPI2* and *CaGPI19* levels, respectively, in these strains.

Finally, we addressed the question of whether *CaGPI2* and *CaGPI19* were activators or repressors of *CaGPI15*. We discovered that simultaneous downregulation of *CaGPI2* and *CaGPI19* in *C*. *albicans* results in downregulation of *CaGPI15* and overexpression of either of them activates *CaGPI15*. In other words, both *CaGPI2* and *CaGPI19* could independently activate *CaGPI15*. Thus, we propose a model for how the three subunits of the GPI-GnT complex interact in *C*. *albicans* (Fig. 5D): *CaGPI15* stimulates activation of *CaGPI2* as well as *CaGPI19*. Downregulating it can simultaneously decrease levels of both *CaGPI2* and *CaGPI19*. In turn, both *CaGPI2* and *CaGPI19* can independently activate *CaGPI15*. Disrupting either *CaGPI2* or *CaGPI19* results in upregulation of the other, which in turn upregulates *CaGPI15*. Both *CaGPI2* and *CaGPI19* are mutually negatively regulated and function downstream of *CaGPI15* as far as hyphal growth and azole drug response are concerned. Sensitivity to azoles *via CaERG11* regulation correlates with *CaGPI19* levels while Ras-dependent hyphal morphogenesis correlates with *CaGPI2* levels.

Two additional issues needed to be addressed here. The first is the mechanism by which *CaERG11* is downregulated in the *CaGPI15* mutant. The presence of Upc2, a transcription factor for *CaERG11*, was found to be reduced on the promoter of the *CaERG11* gene in *CaGPI15* deficient strain. In exploring the possible reason for this, it was observed that H3Ac on the promoter of *CaERG11* is also reduced in the azole sensitive *CaGPI15* mutant strain. Since *CaGPI19* functions downstream of *CaGPI15*, this strain was also tested for H3Ac and found to have reduced levels of it on the *CaERG11* promoter. This is specifically due to loss of Rtt109 activity in these strains. Overexpressing *RTT109* could restore the levels of the GPI-GnT subunits as well as of *CaERG11* and reverse the response to azoles for both *CaGPI15* and *CaGPI19* mutant strains.

The second is the mechanism by which Ras signaling is altered in the *CaGPI15* mutant. Overexpressing *CaRAS1*, but not *CaRAS2*, could restore filamentation in *CaGPI15* mutant. Further, *CaGPI2* functions downstream of *CaGPI15* in this. In a recent manuscript, we showed that CaGpi2 physically interacts with CaRas1 in the endoplasmic reticulum and this interaction helps CaRas1 activate GPI-GnT activity^17^. CaGpi2 also regulates Ras signaling that occurs at the plasma membrane to trigger hyphal morphogenesis, but it does so by its effect on Hsp90 levels in the cell. It is well known that Hsp90 along with its co-chaperone, Sgt1, interacts with Cyr1, the effector of CaRas1, thereby preventing the interaction of GTP-bound CaRas1 with Cyr1 for initiating cAMP-dependent hyphal morphogenesis^32^. Overexpressing *CaGPI2* results in downregulation of Hsp90 and a lifting of the inhibition exerted by Hsp90 on the Ras signaling pathway. Since CaGpi2 seems to control hyphal morphogenesis in the *CaGPI15* mutant strain, it is reasonable to expect that this mechanism functions here too.

The *CaGPI15* heterozygous mutant was also far more susceptible to killing by macrophages and epithelial cells in our assays. It showed reduced internalization by epithelial cells, and also shorter hyphae length in comparison to BWP17. In addition, its ability to damage macrophages and epithelial cells was significantly lesser than that of the wild type strain. The damage caused to macrophages and epithelial cells by infection with BWP17 and *CaGPI15* heterozygous mutant involved apoptosis as well as pyroptosis, but significantly the pyroptosis seen in epithelial cells on infection with *CaGPI15* heterozygous mutant was only 30% of that seen on infection with BWP17. This could be an important contributing factor in reduced damage to epithelial cells by this mutant. Hence, we infer that the *CaGPI15* mutant strain is attenuated in virulence.

In conclusion, what is evident from our previous and current studies is that alterations in levels of different subunits of the same complex, all of which affect GPI biosynthesis, receive varying responses from ergosterol biosynthesis and hyphal filamentation pathways in *C*. *albicans*. Given the importance of GPI biosynthesis for the viability and growth of *C*. *albicans*, the multiple modes of interaction and regulation probably allow the GPI biosynthetic pathway to rapidly respond to multiple signals.

The clinical implications of these observations are hard to miss. Hyphal filamentation and invasive growth are important for the establishment of infection by *C*. *albicans* and several hyphae-specific factors are known to be GPI anchored^7,8^. Strains with reduced levels of GPI anchored proteins are known to show attenuated virulence^8^. Additionally, ergosterol and its biosynthetic pathway continue to be the most important targets of the currently available antifungals. However, one of the major problems in treatment of fungal infections has been the rapid drug resistance that develops upon continued usage of these drugs^33^. CaGpi15 as an alternative target, a master regulator that simultaneously affects hyphal morphogenesis as well as ergosterol biosynthesis could be an interesting candidate. Indeed, strains in which *CaGPI15* levels are reduced are less virulent, as shown here. Additionally, GPI biosynthesis itself is an essential pathway in *C*. *albicans* and targeting the first step of GPI biosynthesis *via* a protein that bears little homology with its mammalian counterpart could be an effective strategy.

## Methods

### Strains and media

Yeast and *C*. *albicans* strains used in this study are described in Table 1. Murine alveolar epithelial type I cell line (LA-4) and Murine alveolar macrophage cell line (MH-S) were procured from the American type tissue culture collection (ATCC), Rockville, MD, USA. All primers used in this study are listed in Supplementary Table 1. Strains were grown in yeast extract-peptone-dextrose (YEPD) media or synthetic dextrose minimal media (SD media). Ura^−^ strains were grown in YEPD or SD media supplemented with 60 µg/ml uridine. Similarly, His^−^ strains or Arg^−^ strains were grown in SD medium supplemented with the appropriate amino acid (85.6 μg/ml His or Arg). Transformations were performed using lithium-acetate method^34^. RPMI-1640 medium was from Sigma, MO, USA and FBS from Gibco, Life technologies, Grand Island, NY, USA. RNA PolII (sc-21750) from Santa Cruz Biotechnology, Histone H3 (mAb-96C10) from Cell Signaling, H3K56Ac (ab71956) from Abcam and H3K9Ac (mAb-H0913) from Sigma.

**Table 1:** List of *C*. *albicans* strains used in the study.

Strains	Genotype	Referred in the manuscript	Reference
YPH500	YPH500	YPH500	^42^
YPH-*pGAL1-ScGPI15*	As YPH500, with *URA3*-*pGAL1*-*ScGPI15*	YPH-*pGAL1-ScGPI15*	This study
YPH-*pGAL1-ScGPI15-CaGPI15*	As YPH-*GAL1-ScGPI15*, with pYEpHIS-*pPMA1*-*CaGPI15*	YPH-*pGAL1-ScGPI15-CaGPI15*	This study
BWP17	*ura3*::imm434*/ura3*::imm434 *iro1/iro1*::imm434 *his1*::hisG*/his1*::hisG *arg4 /arg4*	BWP17	^15^
BWP17URA3	BWP17 with *CaRPS1*/*Carps1Δ::pACT1 CaGFP::URA3*	BWP17URA3	This study
*Cagpi15*/*CaGPI15*	BWP17 with *CaGPI15/Cagpi15Δ::HIS1*	*CaGPI15Hz*	^17^
*Cagpi2/CaGPI2*	BWP17 with *CaGPI2/Cagpi2Δ::ARG4*	*CaGPI2Hz*	^13^
*Cagpi19/CaGPI19*	BWP17 with *CaGPI19/Cagpi19Δ::HIS1*	*CaGPI19Hz*	^11^
*Cagpi15/CaGPI15*-*URA3*	BWP17-*CaGPI15* heterozygous with *CaRPS1/Carps1Δ::pACT1-CaGFP::URA3*	*CaGPI15Hz-URA3*	^17^
*Cagpi15*/*pMET3*-*CaGPI15*	BWP17-*CaGPI15* heterozygous with *pMET3-CaGPI15::URA3*	*Cagpi15 null*	This study
*Cagpi15/CaGPI15/pACT1*-*CaGPI15*	BWP17-*CaGPI15* heterozygous with *CaRPS1/Carps1Δ::pACT1-CaGPI15*	*CaGP15* revertant	^17^
*Cagpi15/CaGPI15::Cagpi2/ CaGPI2*	BWP17-*CaGPI15* heterozygous with *CaGPI2/Cagpi2Δ::URA3*	*CaGPI15Hz/CaGPI2Hz*	This study
*Cagpi15/CaGPI15/pACT1-CaGPI2*	BWP17-*CaGPI15* heterozygous with *CaRPS1/Carps1Δ::pACT1-CaGPI2*	*CaGPI15Hz/pACT1-CaGPI2*	^17^
*Cagpi15/CaGPI15::Cagpi19/CaGPI19*	BWP17-*CaGPI15* heterozygous with *CaGPI19/Cagpi19Δ::ARG4*	*CaGPI15Hz/CaGPI19Hz*	This study
*Cagpi15/CaGPI15/pACT1*-*CaGPI19*	BWP17-*CaGPI15* heterozygous with *CaRPS1/Carps1Δ::pACT1-CaGPI19*	*CaGPI15Hz/pACT1-CaGPI19*	This study
*Cagpi15/CaGPI15/pACT1*-*CaRAS1*	BWP17-*CaGPI15* heterozygous with *CaRPS1/Carps1Δ::pACT1-CaRAS1*	*CaGPI15Hz/pACT1-CaRAS1*	This study
*Cagpi15/CaGPI15/pACT1*-*CaRAS2*	BWP17-*CaGPI15* heterozygous with *CaRPS1/Carps1Δ::pACT1-CaRAS2*	*CaGPI15Hz/pACT1-CaRAS2*	This study
*Cagpi15/CaGPI15/pACT1*-*RTT109*	BWP17-*CaGPI15* heterozygous with *CaRPS1/Carps1Δ::pACT1-RTT109*	*CaGPI15Hz/pACT1-RTT109*	This study
*Cagpi19/CaGPI19*-*URA3*	BWP17-*CaGPI19* heterozygous with *CaRPS1/Carps1Δ::pACT1-CaGFP::URA3*	*CaGPI19Hz-URA3*	^17^
*Cagpi19/CaGPI19*::*Cagpi2/CaGPI2*	BWP17-*CaGPI19* heterozygous with *CaGPI2/Cagpi2Δ::URA3*	*CaGPI19Hz/CaGPI2Hz*	^17^
*Cagpi19/CaGPI19/pACT1*-*RTT109*	BWP17-*CaGPI19* heterozygous with *CaRPS1/Carps1Δ::pACT1-RTT109*	*CaGPI19H/pACT1-RTT109*	This study
*Cagpi2/CaGPI2::Cagpi19/CaGPI19*	BWP17-*CaGPI2* heterozygous with *CaGPI19/Cagpi19Δ::HIS1*	*CaGPI2Hz/CaGPI19Hz*	^13^
BWP17/*pACT1*-*CaGPI19*	BWP17 with *CaRPS1*/*Carps1Δ::pACT1-CaGPI19*	BWP17/*pACT1*-*CaGPI19*	This study
BWP17/*pACT1*-*CaGPI2*	BWP17 with *CaRPS1*/*Carps1Δ::pACT1-CaGPI2*	BWP17/*pACT1*-*CaGPI2*	^17^
BWP17/*pACT1*-*CaGPI15*	BWP17 with *CaRPS1*/*Carps1Δ::pACT1-CaGPI15*	BWP17/*pACT1*-*CaGPI15*	This study
BWP17/*pACT1*-*RTT109*	BWP17 with *CaRPS1*/*Carps1Δ::pACT1-RTT109*	BWP17/*pACT1*-*RTT109*	This study
*Cavps75/CaVPS75*-*URA3*	BWP17 with *CaVPS75/Cavps75Δ::ARG4* with *CaRPS1/Carps1Δ::pACT1-CaGFP::URA3*	*CaVPS75Hz-URA3*	This study

### Chemicals

Chemicals were purchased from Merck, Qualigens, or Sigma-Aldrich (USA) unless specified otherwise. Enzymes were from Bangalore Genei (India), MBI Fermentas (USA) or New England Biolabs (USA). Gel extraction and PCR purification kits were from Qiagen. Primers were synthesized by GCC Biotech (India).

### PCR amplification of *CaGPI15* gene

The putative *CaGPI15* gene sequence was obtained from Prof. Eisenhaber’s web site (http://mendel.imp.ac.at/SEQUENCES/gpi-biosynthesis/pigs-main.html) and also confirmed by BLASTp analysis using the mammalian PIG-H sequence as query. Forward primer FPCaGPI15-pCaDis and reverse primer RPCaGPI15-pCaDis (Supplementary Table 1) were used to amplify *CaGPI15* gene from genomic DNA of *C*. *albicans*. The amplified product was visualized on 1% agarose gel.

### Chromosomal disruption of *CaGPI15* to make heterozygous *CaGPI15* mutant

*CaGPI15* heterozygote in BWP17 strain of *C*. *albicans* (*CaGPI15Hz*) was made by PCR-mediated disruption using *HIS1* as a selection marker^15,17^.

### Construction of regulatable null mutant of *CaGPI15* gene

Conditional null mutant of *CaGPI15* in BWP17 strain (*Cagpi15 null*) was made by placing the functional gene in *CaGPI15Hz* under the *MET3* promoter using PCR mediated approach^16^.

### Complementarity between *C*. *albicans* and *S*. *cerevisiae* genes

*ScGPI15* gene in *S*. *cerevisiae* YPH500 strain was placed under the glucose responsive *GAL1* promoter by a PCR-mediated approach^11,35^. The transformed colonies were selected on SDUra^−^ plates and confirmed with locus specific PCR amplification. YPH-*pGAL1*-*ScGPI15* was unable grow on glucose media but could easily grow on media containing 4% galactose and 1.5% sucrose. For complementation studies, *CaGPI15* gene, placed under the constitutive *PMA1* promoter, was cloned between *BamHI* and *MluI* site into YEpHIS plasmid and used to transform this strain^36^.

### Preparation of microsomes from yeast and GPI-GnT assay

Primary cultures of yeast strains were grown for 16 h in 4% (w/v) galactose. It was inoculated into 250 ml SD medium containing either 4% galactose or 2% glucose at 30 °C till OD_600nm_ of 2.0 was reached. The cells were harvested and microsomes prepared from it as described previously^37^.

GPI-GnT activity of the microsomes was assayed as described previously^37^. The assay produces both [6^3^H]GlcNAc-PI (*N*-acetyl glucosaminylphosphatidylinositol) and [6^3^H]GlcN-PI (glucosaminylphosphatidylinositol, the product of the next step of the pathway) and were detected using Bioscan AR2000 TLC scanner. The sum of the radioactive counts detected for the two products in the case of the control strain was considered to be 100% and GPI-GnT activity of all strains was calculated relative to it. In all cases averages of data from independent experiments done twice in duplicates along with standard deviations are plotted.

### Preparation of microsomes from *C*. *albicans* and GPI-GnT assay

Microsomes from *C*. *albicans* were prepared using a protocol previously standardized in our lab^13^ with minor modifications^17^. GPI-GnT assay was performed using microsomes corresponding to ~1500 μg protein as described previously^37^. Comparable amount of heat-killed microsomes were used as the negative control for the assay.

### Generating the *CaGPI15* revertant strain

*CaGPI15* revertant strain was previously reported^17^. The *pACT1-CaGPI15* construct was used to transform *CaGPI15Hz* cells and the positive clones were confirmed by PCR. The control strains, BWP17URA3 and *CaGPI15Hz*-*URA3* were previously reported^17^.

### Construction of other overexpression strains in the heterozygote mutants

*CaGPI15Hz*/*pACT1*-*CaGPI2* strain was previously reported^17^. Using the same strategy, plasmid, *pACT1-CaGPI19* was used to generate *CaGPI15Hz*/*pACT1*-*CaGPI19*. Plasmid *pACT1-CaRAS1* or *pACT1-CaRAS2* was used to generate *CaGPI15Hz*/*pACT1*-*CaRAS1* and *CaGPI15Hz*/*pACT1*-*CaRAS2*, respectively. The plasmid *pACT1-RTT109* was used to generate *CaGPI15Hz*/*pACT1*-*RTT109* and *CaGPI19Hz*/*pACT1*-*RTT109* strains. BWP17/*pACT1*-*CaGPI2* strain was previously reported^17^. The overexpression strains BWP17/*pACT1*-*CaGPI19* and BWP17/*pACT1*-*CaGPI15* were similarly made. The control strain *CaGPI19Hz*-*URA3* was previously reported^17^. Positive colonies were confirmed by PCR using appropriate primers (Supplementary Table 1).

### Generation of double heterozygous strains

*CaGPI15Hz/CaGPI19Hz* and *CaGPI15Hz/CaGPI2Hz* were generated using the strategy previously described^13,17^. Confirmation was done by using gene-flanking primers (Table 1).

### Generation of *VPS75Hz* strain

*CaVPS75Hz* in BWP17URA3 strain of *C*. *albicans* (*CaVPS75Hz-URA3*) was made by PCR-mediated disruption using *ARG4* as a selection marker. Confirmation was done by using gene-flanking primers (Table 1).

### Monitoring growth rate in liquid cultures

Growth rate of different mutants was monitored by plotting growth curves. Primary cultures were grown overnight in liquid SD medium at 30 °C, 220 rpm. Secondary cultures were set to an initial OD_600nm_ of 0.2 in 60 ml fresh medium and subjected to shaking at 220 rpm, 30 °C. OD_600nm_ was measured at every 2 h interval to assess the growth in each strain till the saturation was reached. Doubling times were calculated by plotting the exponential growth phase of the cells between 4 and 10 h (OD_600nm_ vs time) for each strain. All the experiments were done twice in duplicates.

### Hyphal induction

Hyphal growth was monitored in liquid and solid media using the method described previously^12,13^. All experiments were done at least thrice in duplicates using independent cultures.

### Azole response of mutants

Sensitivity of the strains to azole drugs was studied by spot assays^12^ as well as by growth in liquid medium in the presence of ketoconazole. Primary and secondary cultures were set as described earlier in ‘Monitoring growth rate in liquid cultures’ section. Final concentration of ketoconazole (0.08 or 0.2 µg/ml) was maintained in 60 ml of 0.2 OD_600nm_ secondary cultures. The way of collecting the samples for growth measurement and calculating the doubling times were same as described earlier in this section. Two independent experiments with duplicates were done for each set of the mutants.

### Estimation of sterol levels

The levels of ergosterol and lanosterol were quantified by GC-MS as described previously^12^. Data in all graphs are averages of two independent experiments done in duplicates along with standard deviations.

### Quantification of transcript levels through RT-PCR

RNA extraction, cDNA preparation and quantification of transcript levels was done as described previously using RT primers (Supplementary Table 1)^12,13,17,38^. Glyceraldehyde-3-phosphate dehydrogenase (*GAPDH*) was taken as an internal control. Data in all graphs are averages with standard deviations of two experiments done in duplicates using independent cultures.

### Cell Clumping Assay

Cells were grown in minimal media at 30 °C, 220 rpm until saturation. Cells from 500 µl culture were pelleted down at 5000 rpm, 5 min, washed in 1X PBS and resuspended in 50% glycerol. A 5 μl cell suspension was spotted on microscopic slides and observed under Nikon Eclipse Ti Microscope. Cells were quantified for studying cell aggregation.

### Calcoflour white (CFW) staining and Congo red (CR) staining in the cell wall

Sample preparation was done as described previously^13^. Cells were stained with 100 µg/ml CFW or 100 µg/ml CR for 30 min at 30 °C, washed thrice with PBS and observed under a Nikon SMZ TiE fluorescence microscope. Fluorescence intensity was quantified using NIS Elements AR Version 4 software.

### Heat sensitivity assays

A 10 min heat shock exposure at 48 °C was given and plate assays done as at 30 °C for 48 h described earlier^13^.

### Chromatin Immunoprecipitation (ChIP) assay

ChIP was carried out as described previously^13^. Briefly, cells were harvested after cross-linking DNA to protein with 1% (v/v) formaldehyde. Glycine (25 mM) was added and cells lysed by glass beads in lysis buffer (50 mM HEPES, pH 7.4; 140 mM NaCl; 1 mM EDTA; 1% Triton X-100; 1 mM PMSF). After sonication the supernatant was incubated overnight with 1 µg anti-RNA PolII or H3K56Ac antibody. ProteinA-CL agarose beads (20 µl) were added. The beads were spun down after 2 h, washed with lysis buffer, high-salt buffer (lysis buffer with 500 mM NaCl), wash buffer (10 mM Tris-Cl, pH 8.0; 250 mM LiCl; 1 mM EDTA; 0.5% NP-40) and Tris-EDTA buffer. Eluates collected in elution buffer (150 µl Tris-EDTA buffer containing 1.0% SDS) were treated with 20 µg proteinase K. DNA was separated using phenol:chloroform (1:1) and precipitated. The samples were analyzed by PCR using primers for specific regions of promoters.

### Cell line and Maintenance of cell line

MH-S and LA-4 cell line were cultured and maintained in RPMI 1640 media, supplement with 25 mM HEPES (Sigma, MO, USA) (N-[2-100 hydroxyethyl]piperazine-N0-[2-ethanesulfonic acid]), 50 µg/ml gentamicin sulfate, 0.05 mM 2-mercaptoethanol, 300 µg/ml L-glutamine and 50 µg/ml and 10% heat inactivate FBS (Gibco, Life technologies, Grand Island, NY, USA) in a humidified atmosphere containing 5% CO_2_ at 37 °C. Cell lines were maintained as adherent cultures and sub cultured by trypsinization. The cells were then harvested by using 0.25% w/v trypsin from bovine pancreas in 10 mM EDTA disodium salt to detach the monolayer. The harvested cells were collected and pelleted down by centrifugation at 240 g for 5 min at 4 °C. The pelleted cells were suspended in 1 ml media and counted for viability by haemocytometer using trypan blue dye.

### Uptake of *C*. *albicans*

#### CFSE labelling of *C*. *albicans*

*C*. *albicans* cells (100 million) were labelled with 10 μM carboxyfluorescein *N*-succinimidyl ester (CFSE) as described previously^17^.

MH-S or LA-4 cells (0.3 million) cultured in 24-well plate were incubated with CFSE labelled BWP17 and *CaGPI15Hz* at different MOI such as 1:1 and 1:5 for 3 h and 18 h at 37 °C in CO_2_ incubator. The cells were trypsinized and then harvested with PBS. The harvested cells were fixed in 2% PFA and the uptake of stained BWP17 and *CaGPI15Hz* by MH-S or LA-4 cells was assessed by using BD FACS Calibur flowcytometer in FL1 channel using Cell Quest software.

### Co-culture assay *in vitro*

Co-culture assay was done as described previously^17^. MH-S cells or LA-4 cells (0.3 million) were incubated with CFSE labelled fungal cells for 3 h at 1:1 or 1:5 MOI. The phagocytosis-independent uptake of fungal cells by MH-S was monitored using 2.5 µg/ml of Cyt D, an inhibitor of actin polymerization^39^ as described previously^17^.

### Confocal microscopy

For visualization of uptake of *C*. *albicans* by MH-S or LA-4 cells, 0.3 million cells were cultured on glass cover slips overnight. Cells were then co-cultured with CFSE-labelled BWP17 and *CaGPI15Hz* at MOI (1:5) for 3 h and 18 h at 37 °C. Cells were then washed, fixed with 2% paraformaldehyde (PFA) followed by washing thrice with quencher (ammonium chloride) and examined using a confocal laser scanning microscope (Olympus FluoView FV1000). Five images each were captured having Z sections (depths 0.1 µm)^17^. The above co-cultured cells of 18 h time point were used to determine the length of hyphae. Nikon NIS element software was used to measure the length of the hyphae.

### Macrophage or epithelial cell mediated killing of BWP17 and *CaGPI15Hz*

The macrophage or epithelial cell mediated killing of *C*. *albicans* was studied as described previously^17^. The number of *C*. *albicans* was determined as CFU/ml = number of colonies x dilution factor / volume of culture plate.

### Detection of pyroptosis through lactate dehydrogenase (LDH) enzymatic assay

LA-4 and MH-S cells were cultured at a concentration of 50,000 cells/100 µl with complete media in a 96 well cell culture plates. After overnight culture, cells were washed with complete medium to remove debris and dead cells. Cells were co-cultured with BWP17 and *CaGPI15Hz* for 3 h and 18 h at different MOI (1:5) at 37 °C in CO_2_ incubator. The co-cultured cells were then subjected for cell cytotoxicity assay. The cell cytotoxicity assay was performed using Cytotoxicity Detection Kit (Pierce^TM^, USA). The standard protocol assay reported here were performed according to the manufacturer’s instructions. The amount of LDH released either in control (Positive control, spontaneous and maximum release) or in the experimental wells was used to calculate the % specific lysis^40^. The % specific lysis was calculated as: % Specific lysis = (Experimental release − Spontaneous release) × 100/(Maximum release − Spontaneous release).

### Assessment of apoptosis

LA-4 and MH-S cells were cultured at a concentration of 0.3 million cells/ml in a 24 well cell culture plates. After overnight culture, cells were washed with complete medium to remove debris and dead cells. Cells were co-cultured with BWP17 and *CaGPI15Hz* for 18 h at MOI (1:5) at 37 °C in CO_2_ incubator. The cells were then washed with PBS to remove BWP17 and *CaGPI15Hz*. The cells were trypsinzed and washed followed by staining with annexin V APC conjugate (Biolegend, San Diego, CA, USA) to assess the apoptotic cells using BD FACS Calibur^41^ and analyzed through Cell Quest Software.

### Statistical significance of data

Unless otherwise stated, statistical significance of the data (*p* value) was calculated in Sigma plot 8.0 and GraphPad Prism 5.0 using Student’s *t*-test. The *p* value > 0.05 is considered not significant and is depicted by n.s., *p* value ≤ 0.05 is depicted by *p value ≤ 0.005 is depicted by **and *p* value ≤ 0.0005 is depicted by***.

## Supplementary information

Supplementary dataset 1

## Data Availability

The datasets and material generated during and/or analysed during the current study are available from the corresponding author on reasonable request.
