# Supplementary material for: Modulation of azole sensitivity and filamentation by GPI15, encoding a subunit of the first GPI biosynthetic enzyme, in Candida albicans
Source: Sci Rep. 2019 Jun 11;9:8508. doi: 10.1038/s41598-019-44919-4 (PMC6559964; doi:10.1038/s41598-019-44919-4)
Supplement: Supplementary file 1 — Supplementary dataset 1 [file 41598_2019_44919_MOESM1_ESM.doc]

Modulation of azole sensitivity and filamentation by *GPI15,* encoding a subunit of the first GPI biosynthetic enzyme, in *Candida albicans*

**Priyanka Jain^1†^, Pramita Garai^1†^, Subhash Chandra Sethi^1^, Nilofer Naqvi^1^, Bhawna Yadav1^, Pravin Kumar^1#^, Sneh Lata Singh^1^, Usha Yadav^1^, Shilpi Bhatnagar^1^, Rahul ^1^, Niti Puri^1^, Rohini Muthuswami^1*^ and Sneha Sudha Komath^1*^**

^1^School of Life Sciences, Jawaharlal Nehru University, New Delhi 110067, India.

^Current Address: Post-doctoral Fellow, Fungal Research Group, Institute of Medical Sciences, University of Aberdeen, UK

^#^Current address: Research associate, National Institute of Plant Genome Research, New Delhi 110067, India

^†^ These authors have contributed equally to this work

^*^ **To whom correspondence should be addressed: Sneha Sudha Komath,** School of Life Sciences, Jawaharlal Nehru University, New Delhi 110067, India; sskomath@mail.jnu.ac.in; ; + 91-11-26704502; Rohini Muthuswami, School of Life Sciences, Jawaharlal Nehru University, New Delhi 110067, India; rohini_m@mail.jnu.ac.in; + 91-11-26704154

**Supplementary Information**

**Supplementary Table 1. List of primers used in the study for making the mutants**.

| FPCaGPI15-HIS1 | 5'-ATGTCCTCATCGAAAAATTATAAATTGGAAATTTCCCCTAGTAGTATCAACCAA  ACTAGTGCCACAGAATCGCGGGGATCCTGGAGGATGAG-3' |
| --- | --- |
| RPCaGPI15-HIS1 | 5'-CTAAATAACTTGTTTTAAACCTTGACCAGGCACTCTTCTCCAATAGCGTTTTGTG  CTTCCAAATAGTAACGGAATATTTATGAGAAACT-3' |
| FPCaGPI15-pCaDis | 5'-GCGGGATCCATGTCCTCATCGAAAAATTATAAA-3' |
| RPCaGPI15-pCaDis | 5'-GGCGTCGACCTAAATAACTTGTTTTAAACCTTGAC-3' |
| FPCaGPI15-URA3-pMET3GFP | 5'-ATTGTTTATAAATCAACAAAAGTTTTACCATTTGTTGGTGTAATTACTTTCTTTT  ATTCTGTTTGTTTTTTCTAGAAGGACCACCTTTGATTG-3' |
| RPCaGPI15-URA3-pMET3GFP | 5'-TCTGCGATTCTGTGGCACTAGTTTGGTTGATACTAGGGGAAATTTCCAATTTATA  ATTTTTCGATGAGGATTTGTACAATTCATCCATAC-3' |
| FPpMET3-BamHI-KpnI | 5'-GCGGGATCCGGTACCAATTGTCTATTCCAAGCCTGTGG-3' |
| FPGFP-BamHI | 5'-GGATCCGCGTTTATTAAAATGTCTAAA-3' |
| RPGFP-HindIII | 5'-AAGCTTGGGCTGCAGTTATTTGTACAA-3' |
| FPURA3 | 5'-GCTCGAGAGCTTCCGGATAATAGGAATTGATT-3' |
| RPURA3 | 5'-GCTCGAGAAGCTTAGAAGGACCACCTTTGAT-3' |
| FPScGPI15 | 5'-GGAAGTGAGAAGAGCCTGAGATTGATCAATAAGAGACATTTATATGCATACATGGGTAA  TAACTGATATAATTAAAT-3' |
| RPScGPI15 | 5'-CTTCTTTCTATTCAGTATACTAGTCTTACCAAATTCATACTCTTTACTAATCATGTTTTTTCT  CCTTGACGTTAAAGT-3' |
| FPCaRAS1-HindIII | 5'GCGAAGCTTATGTTGAGAGAATATAAATTA-3' |
| FPCaRAS2-HindIII | 5' GCGAAGCTTATGTCATTACTACTAGATAGC-3' |
| RPCaRPS1 | 5'-AATAGAGAGAAACTATATTATACAC-3' |
| FPCaGPI2-URA3 | 5'-ATGGAAGAAATACATATAAGCTCTTCCATTTTGCAAGATAGTGATTCAGCTCCCC  CATAATAGGAATTGATTTGGATGG-3' |
| RPCaGPI2-URA3 | 5'-TCAGCTTTGTATACTTGACTTCATTAACTTTGGTTTCGCCACATCCCATGGTCCTTGTTATA ATTGGCCAGTTTTTTTC-3' |
| FPCaGPI2-HindIII | 5'-GCGAAGCTTATGGAAGAAATACATATAAGCTCT-3' |
| RPCaGPI2-NheI | 5'-GCGGCTAGCTCAGCTTTGTATACTTGACTTCAT-3' |
| FPCaGPI19-ARG4 | 5'-ATGATATTCCATTTTAACCAAAAAGAGAAAGCAGATTCAAGTAAATGTAAAACA  ATACATGTGGAATTGTGAGCGGAAG-3' |
| RPCaGPI19-ARG4 | 5'-TCATTCATATAGAACATCATTCACTAATGTAATAGGCAAATCCCAAACACCACTTGGTGCC  TTTCCCAGTCACGACGTT-3' |
| FPCaGPI19-5' Flanking | 5'-TATTCTTTAGAGGATCAAGAAAACAATTGG-3' |
| RPCaGPI19-MluI | 5'-ACGCGTTCATTCATATAGAACATCATTCAC-3' |
| FPCaGPI15-ARG4 | 5'-ATGTCCTCATCGAAAAATTATAAATTGGAAATTTCCCCTAGTATCAACCAAACTA  GTGCCACAGAATCGTGTGGAATTGTGAGGGGAAG-3' |
| RPCaGPI15-ARG4 | 5'-CTAAATAACTTGTTTTAAACCTTGACCAGGCACTCTTCTCCAATAGCGTTTTGTGCTTCCA  AATAGTAATTTCCCAGTCACGACGTT-3' |
| FPCaGPI15 | 5'-GCGGGATCCATGTCCTCATCGAAAAATTATAAA-3' |
| Flanking FPCaGPI15 | 5'-TGGTAACCATTCATCAATCATATAG-3' |
| Flanking RPCaGPI15 | 5'-GGTTTATACATAGATTTTATATACATTG-3' |
| FPRTT109-HindIII | 5'-GCGAAGCTTGATTCTTAAAATCGCGTCCTTTCA-3' |
| RPRTT109-NheI | 5'-GCGGCTAGCCTATTTTGATTTTCTATAATTACT-3' |
| RT FPCaGAPDH | 5'-CAGCTATCAAGAAAGCTTCTG-3' |
| RT RPCaGAPDH | 5'-GATGAGTAGCTTGAACCCAA-3' |
| RT FPCaERG11 | 5'-GTGGTGGTAGACATAGATGT-3' |
| RT RPCaERG11 | 5'-CCATCAATAGTCCATCTTAAA-3' |
| RT FPCaGPI15 | 5'-CCACGATTATGGGCAGGTTA-3' |
| RT RPCaGPI15 | 5'-CGCGGTAAGAATTCTGGAAA-3' |
| RT FPCaGPI2 | 5'-GGCCAATAGCATTTCTAAC-3' |
| RT RPCaGPI2 | 5'-CCATCACAAAAACAGACAAA-3' |
| RT FPCaGPI19 | 5'-CAAGAAGAAGAAGAAGGAGAA-3' |
| RT RPCaGPI19 | 5'-AAACACCACTTGGTGCCTTA-3' |
| RT FPRTT109 | 5'-TCGTTGATTGGATGCTGTAAGG-3' |
| RT RPRTT109 | 5'-ACCAGCTTCAACAGGTTCATAA-3' |
| RT FPCaACT1 | 5'-AGCCCAATCCAAAGAGGTA-3' |
| RT RPCaACT1 | 5'-GCTTCGGTCAACAAAACTGG-3' |
| RT FPCaUBC13 | 5'-TCGGTCCTAATCAATCACCTT-3' |
| RT RPCaUBC13 | 5'-TCTTTCAACACATCCAAACAAA-3' |
| RT FPCaERG3 | 5'-GAGAAGTTGAAGGTAAAGTT-3' |
| RT RPCaERG3 | 5'-CTATATCATCAAAATTGGAA-3' |
| RT FPCaERG4 | 5'-TTGACTGATGGGTGGTACGTTT-3' |
| RT RPCaERG4 | 5'-AAAGCCCAAGTGAATGTTTGAA-3' |
| RT FPCaGPI8 | 5'-TTCAAATAACTGGGCCGTCT-3' |
| RT RPCaGPI8 | 5'-CTGGGAAAGCATTTCTTGGA-3' |
| RT FPCaGPI12 | 5'-ATGTTTTTCGCTCCAT-3' |
| RT RPCaGPI12 | 5'-TGCCAAGTTTCATTCATACCA-3' |
| RT FPCaGPI14 | 5'-GTTGGATTGAACAAAGTCATTAC-3' |
| RT RPCaGPI14 | 5'-TGGCTTGTGAAATGATCCACA-3' |
| FPCaGPI15-HIS1 | 5'-ATGTCCTCATCGAAAAATTATAAATTGGAAATTTCCCCTAGTAGTATCAACCAA  ACTAGTGCCACAGAATCGCGGGGATCCTGGAGGATGAG-3' |
| RPCaGPI15-HIS1 | 5'-CTAAATAACTTGTTTTAAACCTTGACCAGGCACTCTTCTCCAATAGCGTTTTGTG  CTTCCAAATAGTAACGGAATATTTATGAGAAACT-3' |
| FPCaGPI15-pCaDis | 5'-GCGGGATCCATGTCCTCATCGAAAAATTATAAA-3' |
| RPCaGPI15-pCaDis | 5'-GGCGTCGACCTAAATAACTTGTTTTAAACCTTGAC-3' |
| FPCaGPI15-URA3-pMET3GFP | 5'-ATTGTTTATAAATCAACAAAAGTTTTACCATTTGTTGGTGTAATTACTTTCTTTT  ATTCTGTTTGTTTTTTCTAGAAGGACCACCTTTGATTG-3' |
| RPCaGPI15-URA3-pMET3GFP | 5'-TCTGCGATTCTGTGGCACTAGTTTGGTTGATACTAGGGGAAATTTCCAATTTATA  ATTTTTCGATGAGGATTTGTACAATTCATCCATAC-3' |
| FPpMET3-BamHI-KpnI | 5'-GCGGGATCCGGTACCAATTGTCTATTCCAAGCCTGTGG-3' |
| FPGFP-BamHI | 5'-GGATCCGCGTTTATTAAAATGTCTAAA-3' |
| RPGFP-HindIII | 5'-AAGCTTGGGCTGCAGTTATTTGTACAA-3' |
| FPURA3 | 5'-GCTCGAGAGCTTCCGGATAATAGGAATTGATT-3' |
| RPURA3 | 5'-gctcgagaagcttagaaggaccacctttgat-3' |
| FPScGPI15 | 5'-GGAAGTGAGAAGAGCCTGAGATTGATCAATAAGAGACATTTATATGCATACATGGGTAA  TAACTGATATAATTAAAT-3' |
| RPScGPI15 | 5'-CTTCTTTCTATTCAGTATACTAGTCTTACCAAATTCATACTCTTTACTAATCATGTTTTTTCT  CCTTGACGTTAAAGT-3' |
| FPCaRAS1-HindIII | 5'GCGAAGCTTATGTTGAGAGAATATAAATTA-3' |
| FPCaRAS2-HindIII | 5' GCGAAGCTTATGTCATTACTACTAGATAGC-3' |
| RPCaRPS1 | 5'-AATAGAGAGAAACTATATTATACAC-3' |
| FPCaGPI2-URA3 | 5'-ATGGAAGAAATACATATAAGCTCTTCCATTTTGCAAGATAGTGATTCAGCTCCCC  CATAATAGGAATTGATTTGGATGG-3' |
| RPCaGPI2-URA3 | 5'-TCAGCTTTGTATACTTGACTTCATTAACTTTGGTTTCGCCACATCCCATGGTCCTTGTTATA ATTGGCCAGTTTTTTTC-3' |
| FPCaGPI2-HindIII | 5'-GCGAAGCTTATGGAAGAAATACATATAAGCTCT-3' |
| RPCaGPI2-NheI | 5'-GCGGCTAGCTCAGCTTTGTATACTTGACTTCAT-3' |
| FPCaGPI19-ARG4 | 5'-ATGATATTCCATTTTAACCAAAAAGAGAAAGCAGATTCAAGTAAATGTAAAACA  ATACATGTGGAATTGTGAGCGGAAG-3' |
| RPCaGPI19-ARG4 | 5'-TCATTCATATAGAACATCATTCACTAATGTAATAGGCAAATCCCAAACACCACTTGGTGCC  TTTCCCAGTCACGACGTT-3' |
| FPCaGPI19-5' Flanking | 5'-TATTCTTTAGAGGATCAAGAAAACAATTGG-3' |
| RPCaGPI19-MluI | 5'-ACGCGTTCATTCATATAGAACATCATTCAC-3' |
| FPCaGPI15-ARG4 | 5'-ATGTCCTCATCGAAAAATTATAAATTGGAAATTTCCCCTAGTATCAACCAAACTA  GTGCCACAGAATCGTGTGGAATTGTGAGGGGAAG-3' |
| RPCaGPI15-ARG4 | 5'-CTAAATAACTTGTTTTAAACCTTGACCAGGCACTCTTCTCCAATAGCGTTTTGTGCTTCCA  AATAGTAATTTCCCAGTCACGACGTT-3' |
| FPCaGPI15 | 5'-GCGGGATCCATGTCCTCATCGAAAAATTATAAA-3' |
| Flanking FPCaGPI15 | 5'-TGGTAACCATTCATCAATCATATAG-3' |
| Flanking RPCaGPI15 | 5'-GGTTTATACATAGATTTTATATACATTG-3' |
| FPRTT109-HindIII | 5'-GCGAAGCTTGATTCTTAAAATCGCGTCCTTTCA-3' |
| RPRTT109-NheI | 5'-GCGGCTAGCCTATTTTGATTTTCTATAATTACT-3' |
| RT FPCaGAPDH | 5'-CAGCTATCAAGAAAGCTTCTG-3' |
| RT RPCaGAPDH | 5'-GATGAGTAGCTTGAACCCAA-3' |
| RT FPCaERG11 | 5'-GTGGTGGTAGACATAGATGT-3' |
| RT RPCaERG11 | 5'-CCATCAATAGTCCATCTTAAA-3' |
| RT FPCaGPI15 | 5'-CCACGATTATGGGCAGGTTA-3' |
| RT RPCaGPI15 | 5'-CGCGGTAAGAATTCTGGAAA-3' |
| RT FPCaGPI2 | 5'-GGCCAATAGCATTTCTAAC-3' |
| RT RPCaGPI2 | 5'-CCATCACAAAAACAGACAAA-3' |
| RT FPCaGPI19 | 5'-CAAGAAGAAGAAGAAGGAGAA-3' |
| RT RPCaGPI19 | 5'-AAACACCACTTGGTGCCTTA-3' |
| RT FPRTT109 | 5'-TCGTTGATTGGATGCTGTAAGG-3' |
| RT RPRTT109 | 5'-ACCAGCTTCAACAGGTTCATAA-3' |
| RT FPCaACT1 | 5'-AGCCCAATCCAAAGAGGTA-3' |
| RT RPCaACT1 | 5'-GCTTCGGTCAACAAAACTGG-3' |
| RT FPCaUBC13 | 5'-TCGGTCCTAATCAATCACCTT-3' |
| RT RPCaUBC13 | 5'-TCTTTCAACACATCCAAACAAA-3' |
| RT FPCaERG3 | 5'-GAGAAGTTGAAGGTAAAGTT-3' |
| RT RPCaERG3 | 5'-CTATATCATCAAAATTGGAA-3' |
| RT FPCaERG4 | 5'-TTGACTGATGGGTGGTACGTTT-3' |
| RT RPCaERG4 | 5'-AAAGCCCAAGTGAATGTTTGAA-3' |
| RT FPCaGPI8 | 5'-TTCAAATAACTGGGCCGTCT-3' |
| RT RPCaGPI8 | 5'-CTGGGAAAGCATTTCTTGGA-3' |
| RT FPCaGPI12 | 5'-ATGTTTTTCGCTCCAT-3' |
| RT RPCaGPI12 | 5'-TGCCAAGTTTCATTCATACCA-3' |
| RT FPCaGPI14 | 5'-GTTGGATTGAACAAAGTCATTAC-3' |
| RT RPCaGPI14 | 5'-TGGCTTGTGAAATGATCCACA-3' |
| FPCaGPI15-HIS1 | 5'-ATGTCCTCATCGAAAAATTATAAATTGGAAATTTCCCCTAGTAGTATCAACCAA  ACTAGTGCCACAGAATCGCGGGGATCCTGGAGGATGAG-3' |
| RPCaGPI15-HIS1 | 5'-CTAAATAACTTGTTTTAAACCTTGACCAGGCACTCTTCTCCAATAGCGTTTTGTG  CTTCCAAATAGTAACGGAATATTTATGAGAAACT-3' |
| FPCaGPI15-pCaDis | 5'-GCGGGATCCATGTCCTCATCGAAAAATTATAAA-3' |
| RPCaGPI15-pCaDis | 5'-GGCGTCGACCTAAATAACTTGTTTTAAACCTTGAC-3' |
| FPCaGPI15-URA3-pMET3GFP | 5'-ATTGTTTATAAATCAACAAAAGTTTTACCATTTGTTGGTGTAATTACTTTCTTTT  ATTCTGTTTGTTTTTTCTAGAAGGACCACCTTTGATTG-3' |
| RPCaGPI15-URA3-pMET3GFP | 5'-TCTGCGATTCTGTGGCACTAGTTTGGTTGATACTAGGGGAAATTTCCAATTTATA  ATTTTTCGATGAGGATTTGTACAATTCATCCATAC-3' |
| FPpMET3-BamHI-KpnI | 5'-GCGGGATCCGGTACCAATTGTCTATTCCAAGCCTGTGG-3' |
| FPGFP-BamHI | 5'-GGATCCGCGTTTATTAAAATGTCTAAA-3' |
| RPGFP-HindIII | 5'-AAGCTTGGGCTGCAGTTATTTGTACAA-3' |
| FPURA3 | 5'-GCTCGAGAGCTTCCGGATAATAGGAATTGATT-3' |
| RPURA3 | 5'-gctcgagaagcttagaaggaccacctttgat-3' |
| FPScGPI15 | 5'-GGAAGTGAGAAGAGCCTGAGATTGATCAATAAGAGACATTTATATGCATACATGGGTAA  TAACTGATATAATTAAAT-3' |
| RPScGPI15 | 5'-CTTCTTTCTATTCAGTATACTAGTCTTACCAAATTCATACTCTTTACTAATCATGTTTTTTCT  CCTTGACGTTAAAGT-3' |
| FPCaRAS1-HindIII | 5'GCGAAGCTTATGTTGAGAGAATATAAATTA-3' |
| FPCaRAS2-HindIII | 5' GCGAAGCTTATGTCATTACTACTAGATAGC-3' |
| RPCaRPS1 | 5'-AATAGAGAGAAACTATATTATACAC-3' |
| FPCaGPI2-URA3 | 5'-ATGGAAGAAATACATATAAGCTCTTCCATTTTGCAAGATAGTGATTCAGCTCCCC  CATAATAGGAATTGATTTGGATGG-3' |
| RPCaGPI2-URA3 | 5'-TCAGCTTTGTATACTTGACTTCATTAACTTTGGTTTCGCCACATCCCATGGTCCTTGTTATA ATTGGCCAGTTTTTTTC-3' |
| FPCaGPI2-HindIII | 5'-GCGAAGCTTATGGAAGAAATACATATAAGCTCT-3' |
| RPCaGPI2-NheI | 5'-GCGGCTAGCTCAGCTTTGTATACTTGACTTCAT-3' |
| FPCaGPI19-ARG4 | 5'-ATGATATTCCATTTTAACCAAAAAGAGAAAGCAGATTCAAGTAAATGTAAAACA  ATACATGTGGAATTGTGAGCGGAAG-3' |
| RPCaGPI19-ARG4 | 5'-TCATTCATATAGAACATCATTCACTAATGTAATAGGCAAATCCCAAACACCACTTGGTGCC  TTTCCCAGTCACGACGTT-3' |
| FPCaVPS75-ARG4 | 5'-GAGAAAGAGATGAGTCAAGTGGAAAGAGAAGCCGAGATCTATAGAATCAAAAAGACACA  ATGTGGAATTGTGAGCGGAAG -3' |
| RPCaVPS75-ARG4 | 5'-AAGCTCTGATTCCTCTACATCATCGTCACTCAAGTCCAATTCTTCACCTTCAGTTGTGTC TTTCCCAGTCACGACGTT-3' |
| FPCaGPI19-5' Flanking | 5'-TATTCTTTAGAGGATCAAGAAAACAATTGG-3' |
| RPCaGPI19-MluI | 5'-ACGCGTTCATTCATATAGAACATCATTCAC-3' |
| FPCaGPI15-ARG4 | 5'-ATGTCCTCATCGAAAAATTATAAATTGGAAATTTCCCCTAGTATCAACCAAACTA  GTGCCACAGAATCGTGTGGAATTGTGAGGGGAAG-3' |
| RPCaGPI15-ARG4 | 5'-CTAAATAACTTGTTTTAAACCTTGACCAGGCACTCTTCTCCAATAGCGTTTTGTGCTTCCA  AATAGTAATTTCCCAGTCACGACGTT-3' |
| FPCaGPI15 | 5'-GCGGGATCCATGTCCTCATCGAAAAATTATAAA-3' |
| Flanking FPCaGPI15 | 5'-TGGTAACCATTCATCAATCATATAG-3' |
| Flanking RPCaGPI15 | 5'-GGTTTATACATAGATTTTATATACATTG-3' |
| FPRTT109-HindIII | 5'-GCGAAGCTTGATTCTTAAAATCGCGTCCTTTCA-3' |
| RPRTT109-NheI | 5'-GCGGCTAGCCTATTTTGATTTTCTATAATTACT-3' |
| RT FPCaGAPDH | 5'-CAGCTATCAAGAAAGCTTCTG-3' |
| RT RPCaGAPDH | 5'-GATGAGTAGCTTGAACCCAA-3' |
| RT FPCaERG11 | 5'-GTGGTGGTAGACATAGATGT-3' |
| RT RPCaERG11 | 5'-CCATCAATAGTCCATCTTAAA-3' |
| RT FPCaGPI15 | 5'-CCACGATTATGGGCAGGTTA-3' |
| RT RPCaGPI15 | 5'-CGCGGTAAGAATTCTGGAAA-3' |
| RT FPCaGPI2 | 5'-GGCCAATAGCATTTCTAAC-3' |
| RT RPCaGPI2 | 5'-CCATCACAAAAACAGACAAA-3' |
| RT FPCaGPI19 | 5'-CAAGAAGAAGAAGAAGGAGAA-3' |
| RT RPCaGPI19 | 5'-AAACACCACTTGGTGCCTTA-3' |
| RT FPRTT109 | 5'-TCGTTGATTGGATGCTGTAAGG-3' |
| RT RPRTT109 | 5'-ACCAGCTTCAACAGGTTCATAA-3' |
| RT FPCaACT1 | 5'-AGCCCAATCCAAAGAGGTA-3' |
| RT RPCaACT1 | 5'-GCTTCGGTCAACAAAACTGG-3' |
| RT FPCaUBC13 | 5'-TCGGTCCTAATCAATCACCTT-3' |
| RT RPCaUBC13 | 5'-TCTTTCAACACATCCAAACAAA-3' |
| RT FPCaERG3 | 5'-GAGAAGTTGAAGGTAAAGTT-3' |
| RT RPCaERG3 | 5'-CTATATCATCAAAATTGGAA-3' |
| RT FPCaERG4 | 5'-TTGACTGATGGGTGGTACGTTT-3' |
| RT RPCaERG4 | 5'-AAAGCCCAAGTGAATGTTTGAA-3' |
| RT FPCaGPI8 | 5'-TTCAAATAACTGGGCCGTCT-3' |
| RT RPCaGPI8 | 5'-CTGGGAAAGCATTTCTTGGA-3' |
| RT FPCaGPI12 | 5'-ATGTTTTTCGCTCCAT-3' |
| RT RPCaGPI12 | 5'-TGCCAAGTTTCATTCATACCA-3' |
| RT FPCaGPI14 | 5'-GTTGGATTGAACAAAGTCATTAC-3' |
| RT RPCaGPI14 | 5'-TGGCTTGTGAAATGATCCACA-3' |
| RT FPCaVPS75 | 5'-CCAATGTATGCCAAAAGACG-3' |
| RT RPCaVPS75 | 5'-AATGACCTGCCTCATCATCG-3' |

**Supplementary Table 2. Growth parameters at 30 ^o^C for all the mutants.** Overnight grown primary cultures diluted to OD_600nm_ of ~0.2 were used to start secondary cultures in growth media as described in the Methods section (the media used are also mentioned in the Table below). Samples were taken after every 2 h and OD_600nm_ was measured. Graphs of OD_600nm_ *vs* time were plotted and doubling times calculated.

| **Strains (Growth Media)** | **Doubling time (in minutes) without ketoconazole** | **Doubling time (in minutes) with ketoconazole** |
| --- | --- | --- |
| BWP17 (SD Ura^+^ Met^-^ Cys^-^) | 126.0 ± 2.08 | 164.7 ± 17.79 |
| *CaGPI15Hz* (SD Ura^+^ Met^-^ Cys^-^) | 124.2 ± 10.15 | 189.5 ± 1.44 |
| *CaGPI15Hz/CaGPI19Hz*  (SD Ura^+^ Met^-^ Cys^-^) | 127.8 ± 7.21 | 213.4 ± 16.75 |
| BWP17URA3 (SD Ura^-^ Met^-^ Cys^-^) | 119.6 ± 2.35 | 167.6 ± 6.22 |
| *CaGPI15Hz-URA3*  (SD Ura^-^ Met^-^ Cys^-^) | 123.1 ± 5.43 | 220.7 ± 8.17 |
| *Cagpi15 null (p)* (SD Ura^-^ Met^-^ Cys^-^) | 113.4 ± 4.33 | 205.9 ± 11.58 |
| *Cagpi15 null (r)* (SD Ura^-^ Met^+^ Cys^+^) | 227.7 ± 7.15 | 262.4 ± 8.48 |
| *CaGPI15 revertant*  (SD Ura^-^ Met^-^ Cys^-^) | 126.8 ± 1.1 | 177.1 ± 2.75 |
| *CaGPI15Hz/pACT1-CaGPI19*  (SD Ura^-^ Met^-^ Cys^-^) | 122.6 ± 195 | 161.6 ± 5.47 |
| *CaGPI19Hz-URA3*  (SD Ura^-^ Met^-^ Cys^-^) | 119.4 ± 0.42 | 219.3 ± 11.24 |
| BWP17*/pACT1-RTT109*  (SD Ura^-^ Met^-^ Cys^-^) | 122.3± 3.47 | 159.3 ± 8.61 |
| *CaGPI15Hz/pACT1-RTT109*  (SD Ura^-^ Met^-^ Cys^-^) | 122.3 ± 5 | 158.1 ± 3.6 |
| *CaGPI19Hz/pACT1-RTT109*  (SD Ura^-^ Met^-^ Cys^-^) | 125.6 ± 2.16 | 163.3 ± 3.22 |

**Supplementary Table 3. Analysis of cell clumping in *CaGPI15* mutants.** The strains were grown overnight at 30 ^o^C until saturation in media conditions described in Methods and then observed under a microscope (Nikon eclipse Ti). A total of 100 cells per strain were counted to obtain an estimate of the tendency of the *CaGPI15* mutants to clump.

| **Strains** | **Single Cells** | **2-5 cells** | **6-10 Cells** | **11-20 cells** | **>21 cells** |
| --- | --- | --- | --- | --- | --- |
| BWP17 | 72 ± 1 | 28 ± 1 | Not Observed | Not observed | Not observed |
| BWP17URA3 | 60 ± 2 | 40 ± 2 | Not Observed | Not Observed | Not observed |
| *CaGPI15Hz-URA3* | 65 ± 1 | 35 ± 1 | Not Observed | Not Observed | Not observed |
| *CaGPI15Hz* | 74 ± 2 | 26 ± 2 | Not Observed | Not Observed | Not observed |
| *Cagpi15 null* | 61 ± 3 | 32 ± 1 | 4 ± 1 | 2 | 1 |
| *CaGPI15 revertant* | 65 ± 1 | 35 ± 1 | Not Observed | Not Observed | Not observed |

**Supplementary figures**


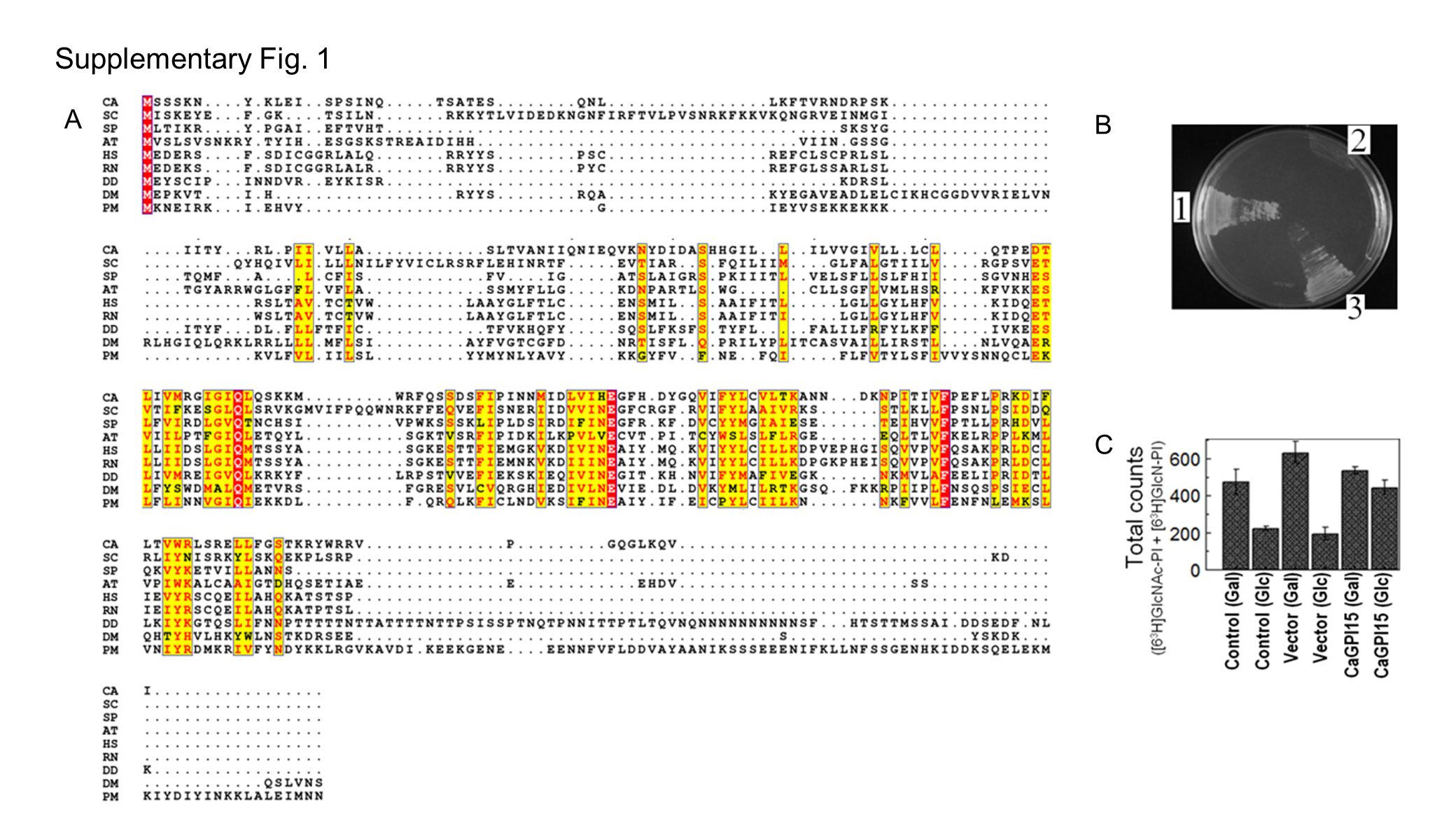


**Supplementary Fig. 1. CaGpi15 is functionally homologous to *S. cerevisiae* Gpi15. (A) Multiple sequence alignment of CaGpi15 with other Gpi15/Pig-H homologs.** The sequence of CaGpi15 was used as query in BLASTp to identify other homologs of the protein. These were then aligned using T-Coffee server and results were generated using ESPript. The residues in yellow box are similar and those in the red box indicate absolute conservation. As can be seen from the figure, there is very little homology between the different homologs of Gpi15 from different organisms. **(B) *CaGPI15* gene can complement growth defect of *S. cerevisiae gpi15* mutant (YPH-*pGAL1-ScGPI15*).** Mutants generated in YPH500 strain background were streaked on YEPD-agar plates. Sector 1: YPH500 wild type cells; Sector 2: YPH-*pGAL1-ScGPI15* wherein the YPH500 strain was transformed with *ScGPI15-URA3-pGAL1-ScGPI15* amplicon. Sector 3: YPH-*pGAL1*-*ScGPI15*-*CaGPI15*; YPH-*pGAL1-ScGPI15* cells were transformed with the episomal plasmid, pPKYEH, constitutively expressing *CaGPI15* gene. **(C) *CaGPI15* can functionally substitute for *ScGPI15* within the GPI-GnT complex.** The GPI-GnT activity in the microsomes of the various strains was measured by the incorporation of [6^3^H]GlcNAc into PI as explained in the Methods section. Control (Glc): YPH-*pGAL1*-*ScGPI15* cells grown in 2% glucose (Glc); Control (Gal): YPH-*pGAL1*-*ScGPI15* cells grown in permissive condition viz. 4% galactose (Gal); *CaGPI15* (Glc): YPH-*pGAL1*-*ScGPI15*-*CaGPI15* cells grown in 2% Glc; Vector (Gal): YPH500 transformed with empty vector; *CaGPI15* (Gal): YPH-*pGAL1*-*ScGPI15*-*CaGPI15* grown in 4% Gal; Control (Gal): YPH-*pGAL1*-*ScGPI15* strain grown in Gal.


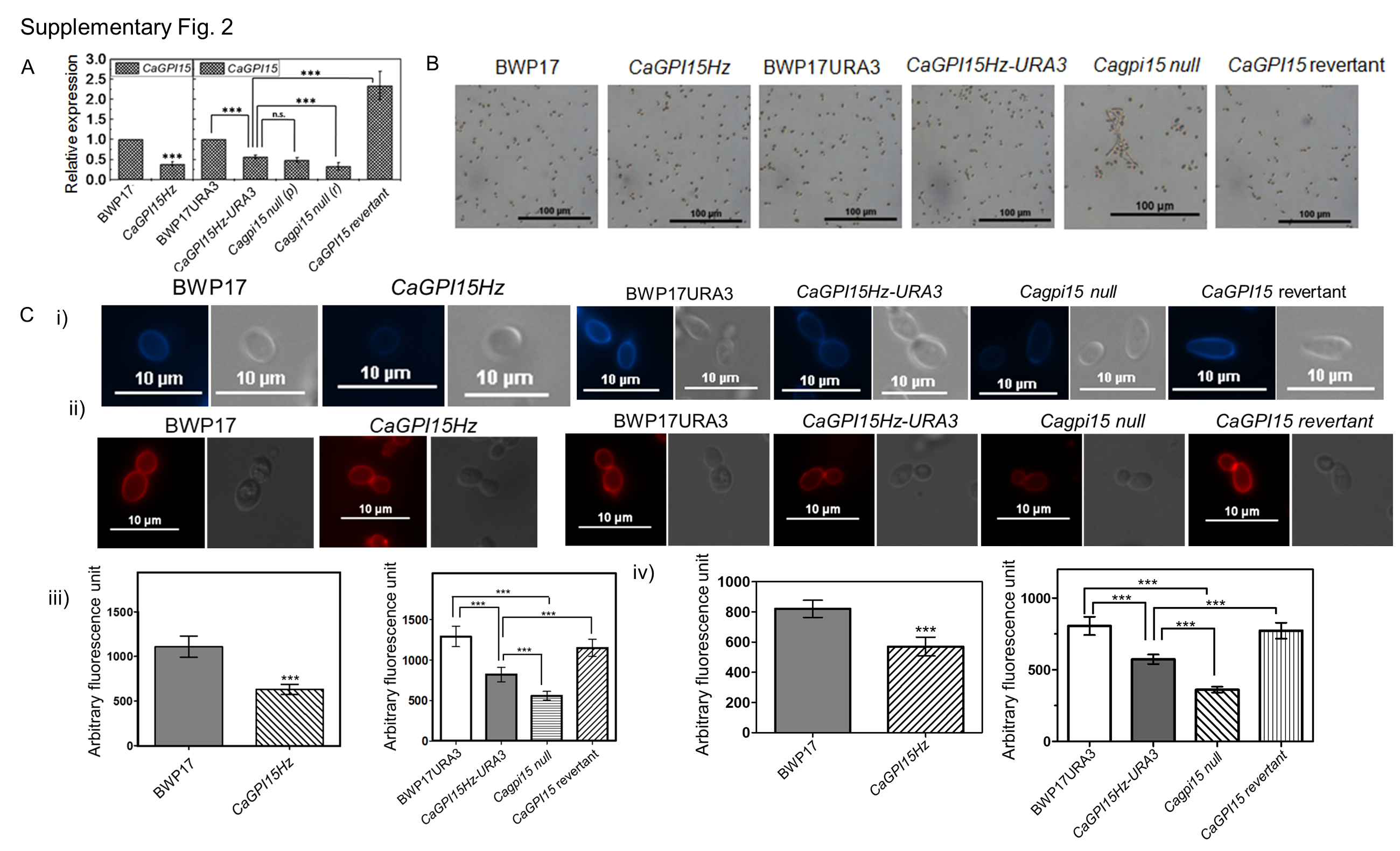


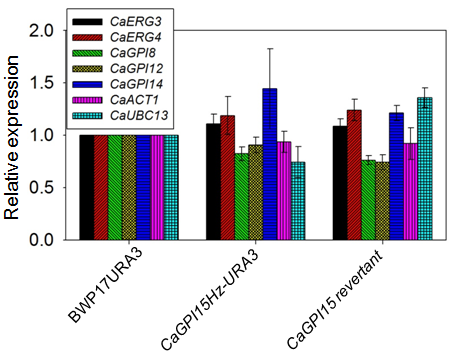
**Supplementary Fig 2. (A) *CaGPI15* transcript levels are down in the *CaGPI15* mutants.** *CaGPI15* transcript levels in *CaGPI15Hz* is plotted relative to BWP17 while in *CaGPI15Hz-URA3* and *CaGPI15* revertant it is plotted relative to BWP17URA. In *Cagpi1*5 *null* transcript levels of *CaGPI15* are shown in the absence (permissive conditions (p)) and in 10 mM Met/Cys (repressive conditions (r)) relative to BWP17URA3. The experiment was done twice in duplicates; averages with standard deviation are plotted. **(B) *CaGPI15* mutants show increased clumping.**  Cell clumping was observed using a Nikon Eclipse Ti Microscope at 20X magnification. **(C) Chitin levels were reduced in the *CaGPI15* mutants.** The chitin levels in the different strains were assessed by **(i)** CFW staining and **(ii)** Congo red staining**.** Quantification of the **(iii)** CFW staining and **(iv)** Congo red staining confirmed the reduction in chitin levels in the *CaGPI15* mutants. A minimum of 100 cells were counted. The experiment was repeated twice. Results from a single experiment done in duplicates along with standard deviations are presented.


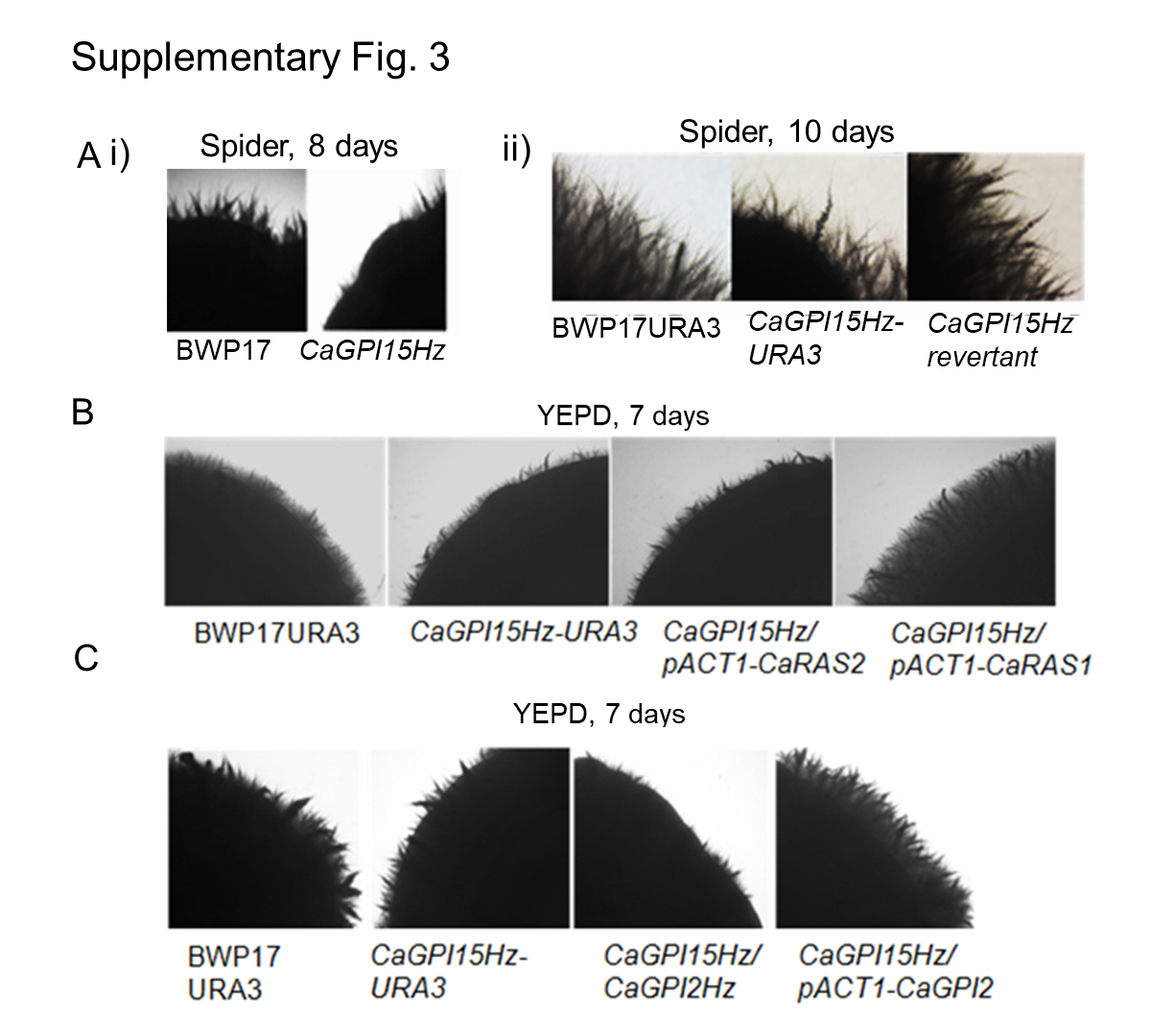


**Supplementary Fig 3. (A) (i) *CaGPI15* is required for filamentation.** The strains were spotted on Spider agar plates and incubated for 8 days at 37 ^o^C. **(ii) Restoration of filamentation in *CaGPI15* revertant mutant**. The strains were spotted on Spider-agar plates and incubated for 10 days at 37 ^o^C to observe hyphal growth. **(B)** **CaRas1 is responsible for the filamentation phenotypes of the *CaGPI15* mutants.** Cells of the indicated strains were spotted on YEPD-agar plates and incubated for 7 days at 37 ^o^C for filamentation. CaRas1 restores filamentation in *CaGPI15Hz.* This experiment was done twice in duplicates. **(C) Hyphal morphogenesis correlates with *CaGPI2* expression levels.** The mutant strains were spotted on YEPD-agar plates and incubated at 37 ^o^C for 7 days along with BWP17URA3 and *CaGPI15Hz*-*URA3* as control strains. The experiment was done thrice in duplicates with independent cultures for confirmation and a representative image is shown.


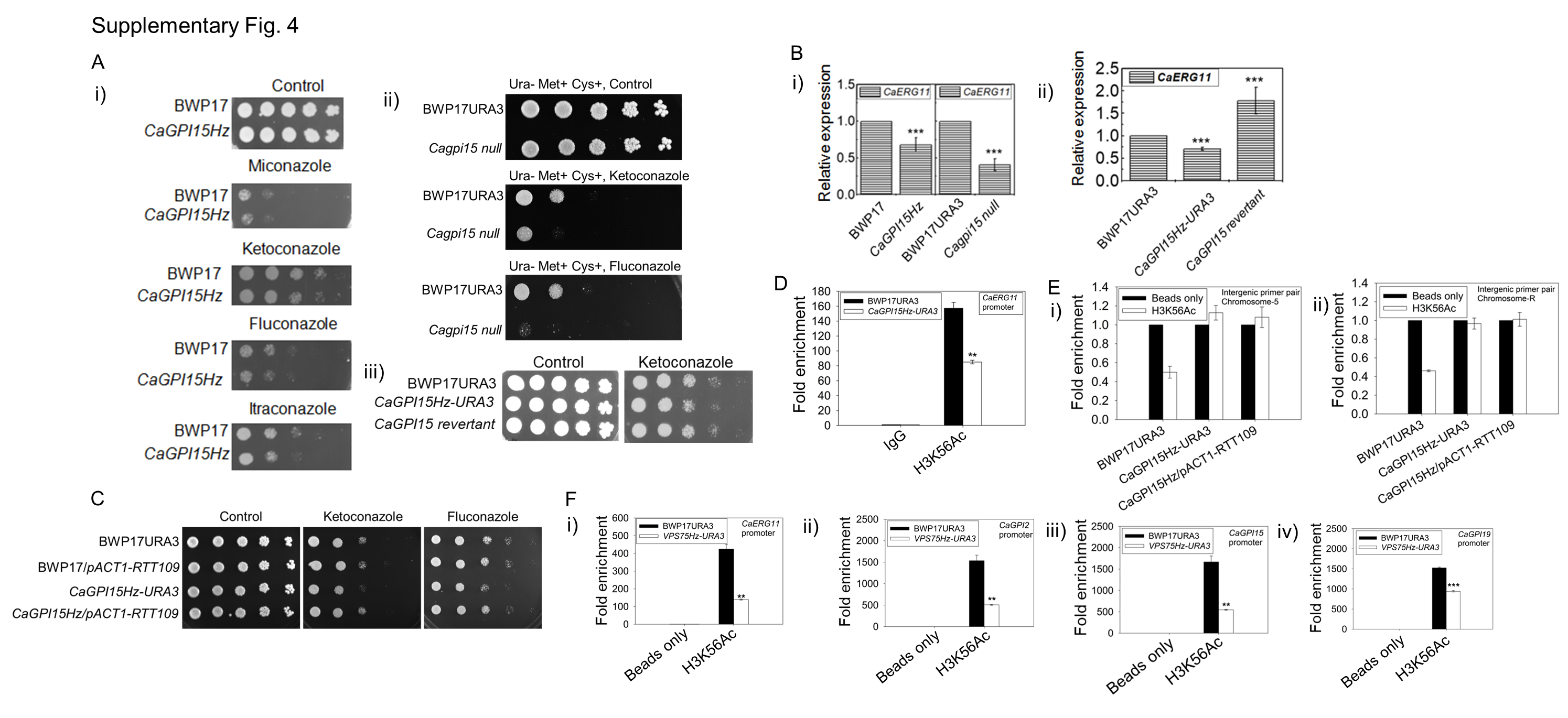


**Supplementary Fig 4. Ergosterol biosynthesis pathway is affected in the *CaGPI15* mutants. (A) The sensitivity of *CaGPI15* mutants to azoles is reversed in the *CaGPI15* revertant. (i)** The strains were spotted on YEPD plates containing miconazole (0.008 μg/ml), ketoconazole (0.2 μg/ml), fluconazole (10.0 μg/ml) or itraconazole (0.5 μg/ml) and incubated at 30 ^o^C for 48 h. **(ii)** *Cagpi15 null* and BWP17URA3 were spotted on SD-agar plates containing either ketoconazole (0.2 μg/ml) or fluconazole (10.0 μg/ml) in the presence of 10 mM Met/Cys and incubated at 30 ^o^C for 72 h. Control plate without azole is also shown. The experiment was repeated thrice using independent cultures. **(iii)** The *CaGPI15* revertant along with controls was spotted on YEPD-agar plate lacking or containing 0.2 µg/ml ketoconazole. Plates were incubated at 30 ^o^C for 24 h. The experiment was done twice. **(B) *CaERG11* levels are specifically downregulated in the *CaGPI15* mutants. (i)** *CaERG11* transcript levels were down in *CaGPI15Hz* and *Cagpi15 null* relative to BWP17 and BWP17URA3, respectively. P-values in all mutants are relative to their respective wild type controls. **(ii)** *CaERG11* transcript levels were restored in *CaGPI15* revertant. P-value in the heterozygous mutant is shown relative to wild type while that in *CaGPI15* revertant is relative to *CaGPI15Hz*-*URA3*. The experiments were done twice in duplicates; averages with standard deviation are plotted. **(C) Azole sensitivity was reversed in *CaGPI15Hz*/*pACT1*-*RTT109* strain**. Strains overexpressing *RTT109* were spotted on YEPD-agar lacking or containing either ketoconazole (0.2 μg/ml) or fluconazole (10.0 μg/ml) and incubated at 30 ^o^C for 72 h. The experiment was done twice in duplicates. **(D) H3K56Ac occupancy on *CaERG11* promoter is decreased in *CaGPI15Hz-URA3* as compared to wild type.** The H3K56Ac level on *CaERG11* promoter (pair-2) region in *CaGPI15Hz-URA3* relative to BWP17URA3. IgG was taken as a negative control. **(E**) **No reduction in H3K56 acetylation in *CaGPI15* mutants at intergenic regions relative to the control** **(i-ii)** The H3K56Ac levels in the intergenic region of Chromosome-5 and Chromosome-R in *CaGPI15Hz-URA3* and *CaGPI15Hz/pACT1*-*RTT109* relative to BWP17URA3. **(F)** **The relative H3K56Ac levels at the promoters of *CaERG11*, *CaGPI2*, *CaGPI15* and *CaGPI19* is decreased in *VPS75Hz* strain compared to wild type** **(i)** The H3K56 Ac levels in the *CaERG11* promoter (pair-2) in *VPS75Hz-URA3* relative to BWP17URA3. **(ii)** The H3K56 Ac levels in the *CaGPI2* promoter (pair-3) in *VPS75Hz-URA3* relative to BWP17URA3. **(iii)** The H3K56 Ac levels in the *CaGPI15* promoter (pair-3) in *VPS75Hz-URA3* relative to BWP17URA3. **(iv)** The H3K56 Ac levels in the *CaGPI19* promoter (pair-3) in *VPS75Hz-URA3* relative to BWP17URA3.


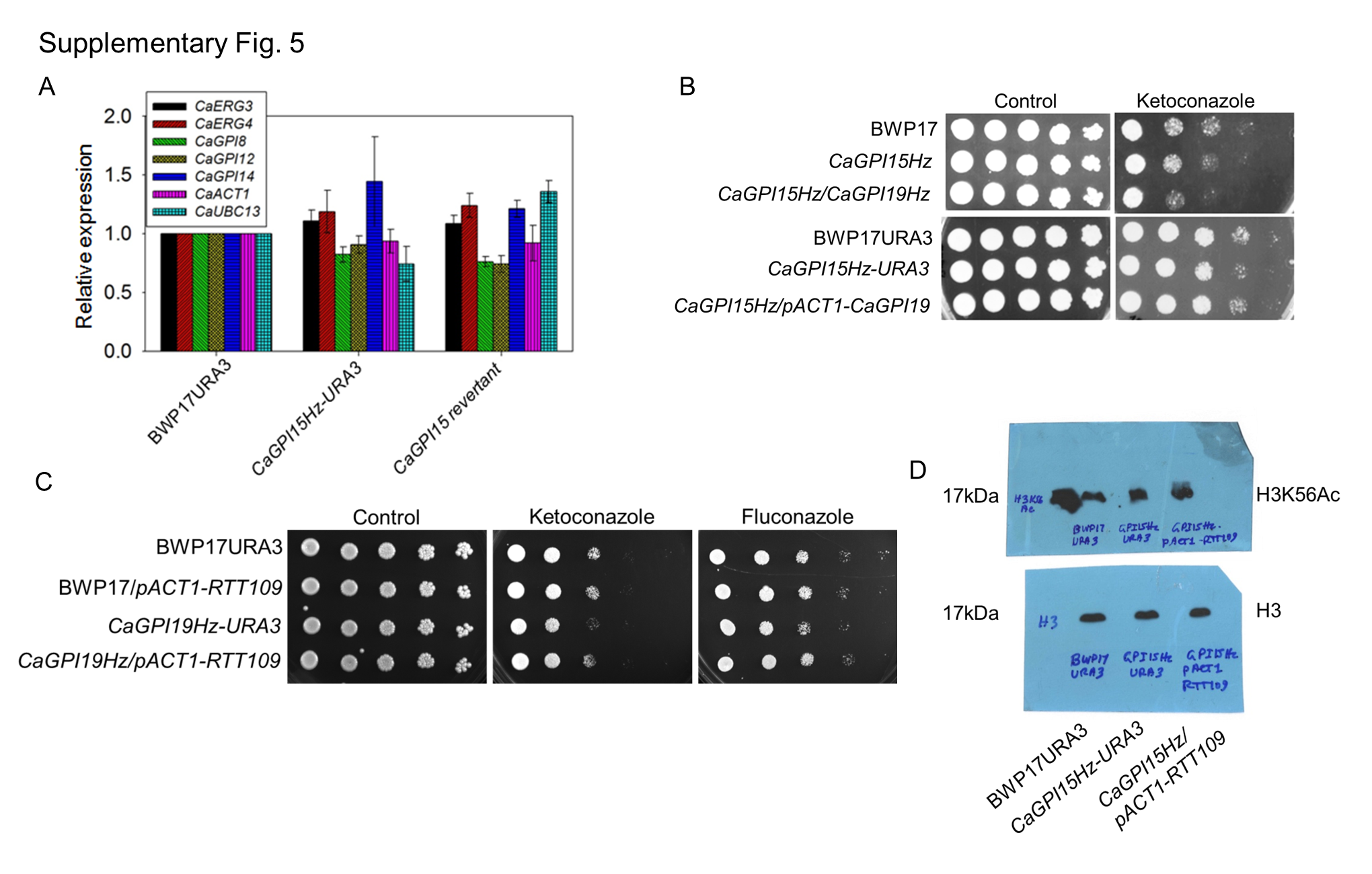


**Supplementary Fig 5. (A) Transcript analysis indicates that a general decrease in transcription does not occur in the *CaGPI15Hz*.** Transcript levels of some housekeeping genes (*CaUBC13, CaACT1*), ergosterol biosynthesis genes *CaERG3, CaERG4*) and downstream GPI biosynthetic genes (*CaGPI12, CaGPI14, CaGPI8*) in BWP17URA3, *CaGPI15Hz* and the *CaGPI15* revertant are shown. **(B) The azole response of the strains correlates with *CaERG11* expression levels.** The strains were spotted on YEPD-agar plates containing ketoconazole (0.2 µg/ml) and incubated at 30 ^o^C. These experiments were done twice in duplicates with independent cultures for confirmation. Representative images are shown. **(C) Rtt109 regulates *CaERG11* transcription and sensitivity to azoles in the *CaGPI19* mutant.** Response to azoles was monitored in *RTT109* overexpression strains relative to their parent strains. The strains were spotted on YEPD-agar plates without or with either ketoconazole (0.2 μg/ml) or fluconazole (10.0 μg/ml) and incubated at 30 ^o^C for 72 h. Two independent experiments were done in duplicates for confirmation. A representative image is shown. **(D)** The full-length blot shows the H3K56Ac levels in whole cell lysate in the mutants relative to BWP17URA3. H3 levels were taken as a loading control.
